# Direct and Indirect Effects of the Coronavirus Disease 2019 Pandemic on Private Healthcare Utilization in South Africa, March 2020–September 2021

**DOI:** 10.1093/cid/ciac055

**Published:** 2022-01-27

**Authors:** Amanda C Perofsky, Stefano Tempia, Jeremy Bingham, Caroline Maslo, Mande Toubkin, Anchen Laubscher, Sibongile Walaza, Juliet R C Pulliam, Cécile Viboud, Cheryl Cohen

**Affiliations:** Fogarty International Center, National Institutes of Health, Bethesda, Maryland, United States; Centre for Respiratory Diseases and Meningitis, National Institute for Communicable Diseases of the National Health Laboratory Service, Johannesburg, South Africa; School of Public Health, Faculty of Health Sciences, University of the Witwatersrand, Johannesburg, South Africa; South African DSI-NRF Centre of Excellence in Epidemiological Modelling and Analysis, Stellenbosch University, Stellenbosch, South Africa; Clinical Division, Netcare Limited, Johannesburg, South Africa; Emergency and Trauma Department, Netcare Limited, Johannesburg, South Africa; Clinical Division, Netcare Limited, Johannesburg, South Africa; Centre for Respiratory Diseases and Meningitis, National Institute for Communicable Diseases of the National Health Laboratory Service, Johannesburg, South Africa; School of Public Health, Faculty of Health Sciences, University of the Witwatersrand, Johannesburg, South Africa; South African DSI-NRF Centre of Excellence in Epidemiological Modelling and Analysis, Stellenbosch University, Stellenbosch, South Africa; Fogarty International Center, National Institutes of Health, Bethesda, Maryland, United States; Centre for Respiratory Diseases and Meningitis, National Institute for Communicable Diseases of the National Health Laboratory Service, Johannesburg, South Africa; School of Public Health, Faculty of Health Sciences, University of the Witwatersrand, Johannesburg, South Africa

**Keywords:** COVID-19, sub-Saharan Africa, healthcare, social distancing, lockdown

## Abstract

**Background:**

The coronavirus disease 2019 (COVID-19) pandemic caused severe disruptions to healthcare in many areas of the world, but data remain scarce for sub-Saharan Africa.

**Methods:**

We evaluated trends in hospital admissions and outpatient emergency department (ED) and general practitioner (GP) visits to South Africa’s largest private healthcare system during 2016–2021. We fit time series models to historical data and, for March 2020–September 2021, quantified changes in encounters relative to baseline.

**Results:**

The nationwide lockdown on 27 March 2020 led to sharp reductions in care-seeking behavior that persisted for 18 months after initial declines. For example, total admissions dropped 59.6% (95% confidence interval [CI], 52.4–66.8) during home confinement and were 33.2% (95% CI, 29–37.4) below baseline in September 2021. We identified 3 waves of all-cause respiratory encounters consistent with COVID-19 activity. Intestinal infections and non–COVID-19 respiratory illnesses experienced the most pronounced declines, with some diagnoses reduced 80%, even as nonpharmaceutical interventions (NPIs) relaxed. Non-respiratory hospitalizations, including injuries and acute illnesses, were 20%–60% below baseline throughout the pandemic and exhibited strong temporal associations with NPIs and mobility. ED attendances exhibited trends similar to those for hospitalizations, while GP visits were less impacted and have returned to pre-pandemic levels.

**Conclusions:**

We found substantially reduced use of health services during the pandemic for a range of conditions unrelated to COVID-19. Persistent declines in hospitalizations and ED visits indicate that high-risk patients are still delaying seeking care, which could lead to morbidity or mortality increases in the future.

## Teaser

We found substantial declines in care seeking at all levels of clinical severity in South Africa’s largest private healthcare system. Non-COVID-19 respiratory illnesses and intestinal infections, followed by injuries, experienced the sharpest reductions and remained below baseline throughout the study.

Worldwide, healthcare systems experienced considerable disruptions during the coronavirus disease 2019 (COVID-19) pandemic, especially in the initial months after the pandemic’s onset in March 2020. Health systems cancelled elective procedures, closed medical practices, and shifted to telemedicine, while public health messaging emphasized avoiding unnecessary healthcare use to reduce exposure to the virus and conserve limited resources. These measures, combined with shelter-in-place orders, resulted in sweeping reductions to hospitalizations, accident and emergency department (ED) attendances, and primary care appointments across a wide spectrum of medical conditions [1–4]. Most of the data, however, come from high-income settings in the United States, Europe, and Asia.

Here, we present a retrospective analysis of the pandemic’s impact on healthcare utilization in South Africa based on the country’s largest private healthcare system. By the end of 2021, South Africa had the largest documented COVID-19 epidemic among African countries, with more than 3.4 million confirmed cases, more than 90 000 laboratory-confirmed deaths, and 280 000 excess deaths from natural causes [5] (85%–95% attributable to COVID-19 [6]). To slow the spread of severe acute respiratory syndrome coronavirus 2 (SARS-CoV-2), South Africa implemented nonpharmaceutical interventions (NPIs) of varying degrees of stringency, including a total lockdown, physical distancing, travel bans, and face mask mandates (Table 1). In December 2021, vaccination remained low, with 26% of the population fully vaccinated [7].

**Table 1.** Timeline of National Coronavirus Disease 2019 Response in South Africa

Lockdown Alert Level	Dates	Restrictions
Pre-lockdown period	1 March 2020–26 March 2020	• March 5: First confirmed coronavirus disease 2019 case • March 15: State of disaster, with closure of all airports, ports, and land crossings • March 18: Schools closed
5	27 March 2020–30 April 2020	• Home confinement, to for essential products/services • Ban on air travel and internal movement • Nonessential services suspended • All gatherings prohibited, to funerals • Sale of alcohol prohibited
4	1 May 2020–31 May 2020	• Some NEB sectors reopen • Restaurants closed, to for off-site consumption • Curfew, 9 PM–4 AM
3	1 June 2020–17 August 2020	• Interprovincial travel permitted • Restaurants reopened, but on-site sale of alcohol prohibited • Face masks required in public places • Gathering limitations: 50 indoors, 100 outdoors • Schools gradually reopened until closure on 27 July • Alcohol ban temporarily lifted until 12 July • Curfew, 10 PM–4 AM
2	18 August 2020–20 September 2020	• No restrictions on internal movement • In-person dining permitted • Gathering limitations: 100 indoors, 250 outdoors • Alcohol ban lifted • Curfew, 11 PM–4 AM
1	21 September 2020–28 December 2020	• Minimal restrictions, but with gathering size limitations and social distancing • NEB sectors, public recreational spaces, and schools reopened • Curfew, 12 AM–4 AM
Adjusted 3	29 December 2020– 28 Februrary 2021	• Closure of schools and public amenities • Alcohol ban, 29 December–1 February • Curfew, 9 PM–6 AM
Adjusted 1	1 March 2021–30 May 2021	• Alcohol ban during Easter weekend • Curfew, 12 AM–4 AM
Adjusted 2	31 May 2021–15 June 2021	• NEB must close by 10 PM • Curfew, 11 PM–4 AM
Adjusted 3	16 June 2021–27 June 2021	• NEB must close by 9 PM • Gathering limitations: 50 indoors, 100 outdoors • Curfew, 10 PM–4 AM
Adjusted 4	28 June 2021–25 July 2021	• Alcohol ban • NEB must close by 8 PM • All gatherings prohibited, except funerals • Schools closed • Nonessential travel to/from Gauteng prohibited • Curfew, 9 PM–4 AM
Adjusted 3	26 July 2021–12 September 2021	• NEB must close by 9 PM • Gathering limitations: 50 indoors, 100 outdoors • No on-site consumption of alcohol after 8 PM • Curfew, 10 PM–4 AM
Adjusted 2	13 September 2021–26 September 2021 (last week of study)	• Partial reopening of borders • Gathering limitations: 250 indoors, 500 outdoors • No on-site consumption of alcohol after 10 PM • Attendance of sporting events prohibited • Curfew, 11 PM–4 AM

Abbreviation: NEB, nonessential business.

South Africa reported its first SARS-CoV-2 infection on 5 March 2020, declared a state of disaster on 15 March, and enforced a national lockdown on 27 March. As in other countries, the government incrementally eased and reinstated NPIs over the subsequent months (based on a 5-level scale). Declines in outpatient and inpatient volumes were documented at the beginning of the pandemic [8–11], but comprehensive, longer-term effects on healthcare use in sub-Saharan Africa have not been reported. We assessed the impacts of COVID-19 and mitigation strategies on healthcare-seeking behavior across diagnoses, age groups, and degrees of clinical severity at different stages of South Africa’s pandemic response during March 2020–September 2021.

## METHODS

### Data Sources

#### Medical Encounters Data

South Africa has a dual health system comprised of ­government-run public hospitals and primary care clinics that serve 84.6% of the population [12] and private health services available to patients who are members of medical aid schemes. Netcare is the largest private network of hospitals, primary healthcare, and emergency medical services in South Africa, representing 25.5% of private hospital beds and spanning all 9 provinces. Netcare and Medicross, their subsidiary network of general practitioners, provided access to weekly numbers of inpatient and outpatient encounters, aggregated by age group  (<5 years, 5–19 years, 20–49 years, ≥50 years, or all ages) and ­diagnosis code, for all patients who sought care during 2016–2021. These data included 16 145 859 total encounters (3 071 189 [19%] inpatient, 2 818 768 [17.5%] outpatient–ED, 10 255 902 [63.5%] outpatient–general practitioner [GP]). Numbers of telehealth consultations at Medicross clinics were available at a monthly resolution (Supplementary Figure 1). The provincial coverage of providers (59 hospitals, 88 clinics) varied according to the type of consultation (Supplementary Figure 2).

Consultations were coded based on discharge diagnosis using the *International Classification of Diseases, 10th revision* (ICD-10). We assessed the impact of the COVID-19 pandemic on respiratory illnesses, systemic chronic conditions that are risk factors for severe COVID-19 disease, COVID-19 complications, and several other conditions that may have been indirectly affected through the lockdown’s effects on care-seeking or physical-distancing (see Supplementary Table 1 for ICD-10 codes and Supplementary Figures 3–5 for weekly time series). Analyses were limited to diagnoses with sufficient levels of reporting (>15 encounters per week), which resulted in different sets of conditions analyzed across consultation types.

The University of Witwatersrand Human Research Ethics Committee approved the study. Deidentified aggregated data were shared; therefore, individual consent was not required.

#### Virologic Surveillance Data

To monitor the circulation of respiratory viruses, we obtained data on the weekly percentage of respiratory samples testing positive for influenza, respiratory syncytial virus (RSV), or SARS-CoV-2 from the outpatient influenza-like illness program and the inpatient pneumonia surveillance program maintained by the National Institute for Communicable Diseases (Supplementary Methods). Data on national COVID-19 testing were obtained from Our World in Data [13].

#### Human Mobility and Government Responses to COVID-19

**Figure 1.**
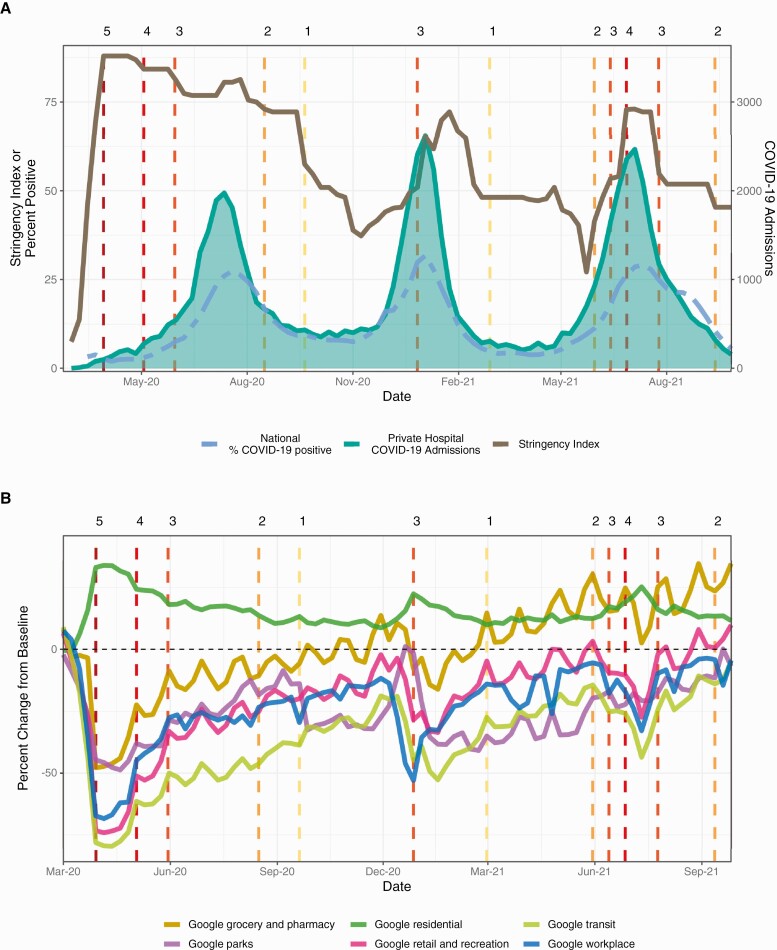
The stringency of government responses to coronavirus disease 2019 (COVID-19) and mobility metrics for South Africa from March 2020 to September 2021. Vertical dashed lines indicate lockdown alert levels (Table 1) and are colored by the stringency of lockdown measures: dark to light. *A,* Weekly time series for the national percentage of positive COVID-19 tests (dashed line), the number of COVID-19–coded admissions in South Africa’s primary private hospital group, and the Oxford policy stringency index for South Africa. *B,* The weekly percent change from baseline for 6 Google mobility categories.

To quantify changes in population behavior, we obtained Google mobility trends [14] for South Africa in 6 location categories (Figure 1, Supplementary Figure 7). To measure variation in South Africa’s policy responses to COVID-19, we used the OxCGRT policy stringency index [15] (Figure 1, Supplementary Figure 6, Supplementary Table 2). See Supplementary Materials for additional details.

### Statistical Analyses

All statistical analyses were conducted using the statistical computing software R version 4.1.0 [16].

We conducted counterfactual analyses in which observed all-cause respiratory consultations were compared to the baseline number of consultations expected in the absence of COVID-19 and NPIs. Analyses were run separately by age and type of consultation: inpatient, outpatient–ED, and outpatient–GP. For national analyses of respiratory conditions, we removed encounters for individuals aged <5 years (117 657 [27%] inpatient, 134 752 [34%] outpatient–ED, 399 839 [14%] outpatient–GP) because they were likely caused by seasonal respiratory virus infections. We fit dynamic regression models with ARIMA errors [17] to weekly numbers of all-cause respiratory consultations from the week of 3 January 2016 to the week of 23 February 2020 and projected the model forward to obtain a baseline for each week of the pandemic period, 1 March 2020 to 26 September 2021. Models were adjusted for seasonality and weekly influenza and RSV activity by including the following as covariates: sine and cosine terms with periods of 52.18 and 26.09 weeks and the weekly percentage of respiratory samples testing positive for influenza or RSV. For the model prediction period, observed weekly percentages of samples testing positive for influenza or RSV were replaced with values from the same epidemic week in 2019. We determined the optimal number of harmonic terms for each time series using Akaike’s information criterion. We conducted similar analyses for each non–COVID-19 diagnosis group. Models for nonrespiratory conditions did not include covariates for influenza and RSV.

Our primary outcome was the weekly percent difference between the observed and predicted incidences, defined as 100 × (observed–predicted)/predicted. We computed 95% prediction intervals using 1000 bootstrap simulations with resampled errors. We measured correlations between the observed number of COVID-19–coded encounters and the weekly percent change from baseline in all-cause respiratory encounters by age and consultation type.

For each diagnosis group, we compared the mean weekly percent change from baseline across 10 phases of the COVID-19 pandemic in South Africa (Table 1).

We used generalized additive models to measure correlations between the weekly percent change from baseline in Google mobility metrics and the weekly percent change from baseline in admissions for each diagnosis group. We compiled the strength of time series cross-correlations and the optimal temporal lag (1–4 weeks) between hospitalizations and visits to residential locations or COVID-19 NPIs from March 2020 to September 2021 (Supplementary Methods).

## RESULTS

### All-Cause Respiratory Encounters During the COVID-19 Pandemic

#### Trends at the National Level and in Adults

**Figure 2.**
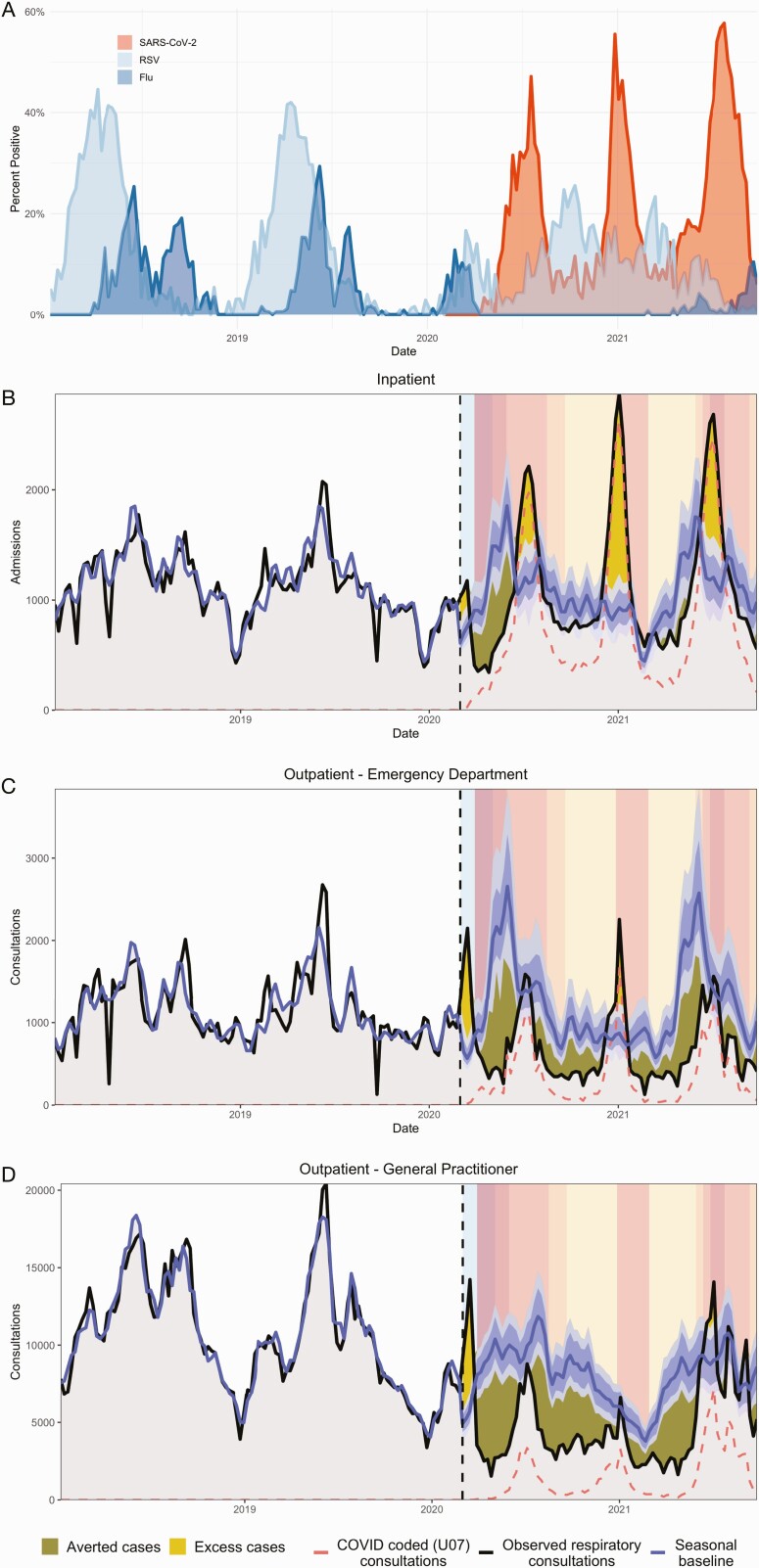
SARS-CoV-2 surveillance and all-cause respiratory encounters in individuals aged ≥5 years. *A,* The percentage of respiratory samples testing positive for influenza, RSV, and SARS-CoV-2 from 2 syndromic respiratory illness surveillance programs in South Africa. *B–D,* Weekly all-cause respiratory consultations (including COVID-19) among individuals aged ≥5 years relative to the baseline number of consultations expected in the absence of COVID-19 at 3 levels of clinical severity: inpatient *(B)*, outpatient–emergency department *(C)*, and outpatient–general practitioner *(D)*. The band is the 95% prediction interval of the projected seasonal baseline. The dashed line is the number of COVID-coded encounters. The vertical dashed line indicates the start of the model prediction period (1 March 2020), and panel colors indicate the pre-lockdown period (1 March 2020–26 March 2020) and lockdown alert levels from March 2020 to September 2021 (Table 1). Panels are shaded according to the stringency of lockdown measures: dark to light. The area between the projected seasonal baseline and observed consultations has dark shading when observed consultations are below baseline (“averted cases”) and light shading when observed consultations are above baseline (“excess cases”). See Supplementary Figure 8 for a closer view of the 2020–2021 time period. Abbreviations: COVID-19, coronavirus disease 2019; RSV, respiratory syncytial virus; SARS-CoV-2, severe acute respiratory syndrome coronavirus 2.

**Figure 3.**
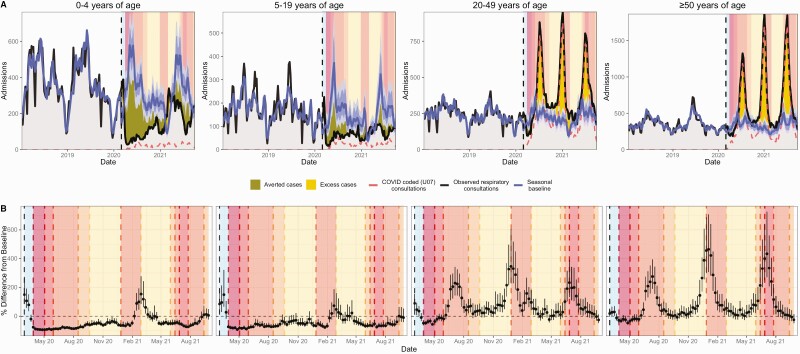
All-cause respiratory admissions in all age groups. *A*, Weekly all-cause respiratory admissions by age group relative to all-cause respiratory admissions expected in the absence of COVID-19. The band is the 95% prediction interval of the projected seasonal baseline. The dashed line is the number of COVID-coded encounters. The vertical dashed line indicates the start of the model prediction period (1 March 2020), and panel colors indicate the pre-lockdown period (1 March 2020–26 March 2020) and lockdown alert levels from March 2020 to September 2021 (Table 1). Panels are shaded according to the stringency of lockdown measures: dark to light. The area between the projected seasonal baseline and observed consultations has dark shading when observed consultations are below baseline (“averted cases”) and light shading when observed consultations are above baseline (“excess cases”). *B,* Weekly observed percent difference from seasonal baseline (95% confidence interval) by age group. Abbreviation: COVID-19, coronavirus disease 2019.

Outpatient all-cause respiratory consultations spiked nationally and across age groups during the week of 15 March 2020 (Figure 2, Supplementary Figures 8–10). After the nationwide lockdown on 27 March 2020 (lockdown level 5), respiratory encounters declined sharply across all consultation types  (Figure 2). Hospitalizations began to climb after shelter-in-place orders eased on 1 May 2020 (level 4), followed by ED and GP encounters after the transition to level 3 on 1 June 2020  (Figures 2 and 3, Supplementary Figures 8–10). Our study period encompassed 3 pandemic waves caused by the ancestral SARS-CoV-2 virus (peak in July 2020), the Beta variant (B.1.351 [18], peak in January 2021), and the Delta variant (B.1.617.2, peak in July 2021). Inpatient and outpatient all-cause respiratory encounters aligned with SARS-CoV-2 viral surveillance and COVID-19–specific encounters (Figures 1–3). In adults aged ≥20 years, the weekly percent change from baseline in all-cause respiratory encounters strongly correlated with COVID-19–coded encounters across all levels of severity (R^2 ^≥ 0.8; Supplementary Figure 11).

#### Trends in Children

After March 2020, all-cause respiratory hospitalizations in children aged <5 years dropped substantially below baseline and continued at low levels until increasing in November 2020 (Figure 3). Admissions in older children gradually increased after the easing of physical distancing restrictions in May 2020. Outpatient respiratory visits in children remained below baseline throughout the pandemic, with the exception of late February 2021, during which both GP visits and admissions spiked (Supplementary Figures 9 and 10).

### National Trends in Non–COVID-19 Encounters

**Figure 4.**
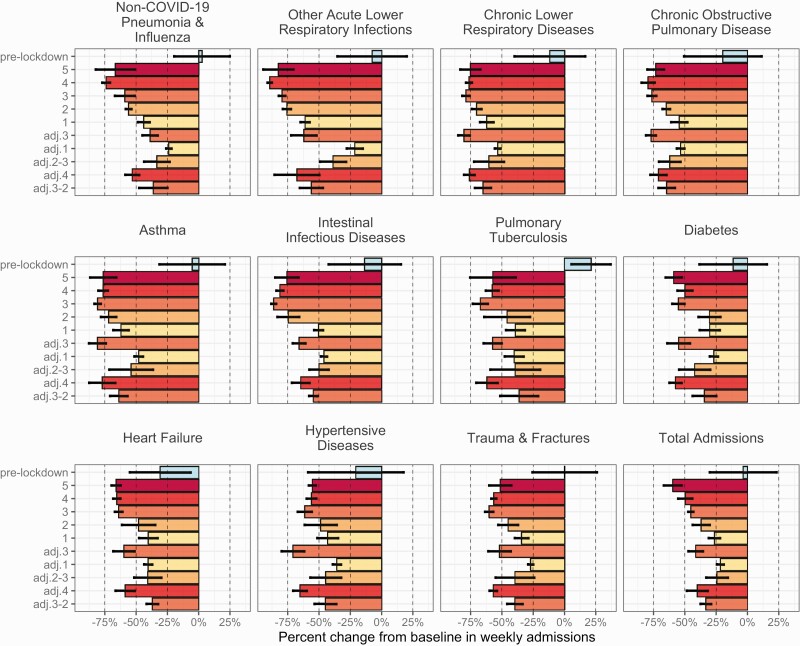
Percent change in inpatient admissions for total admissions and non–COVID-19 diagnoses relative to baseline numbers expected in the absence of COVID-19. The average percent change from baseline in weekly admissions during 10 phases of the COVID-19 pandemic in South Africa: pre-lockdown (1 March 2020–26 March 2020) and lockdown alert levels from March 2020 to September 2021 (Table 1). Bars are shaded according to the stringency of lockdown measures: dark to light. The vertical dashed line indicates a 50% reduction relative to the projected baseline number of admissions. See Supplementary Figure 11 for plots of all diagnosis groupings analyzed in the study. Abbreviation: COVID-19, coronavirus disease 2019.

From 1 March 2020 to the lockdown on 27 March 2020, weekly inpatient admissions were equivalent to or slightly above their projected baselines (Supplementary Table 3). During the strictest lockdown phases (levels 5 to 4), hospitalizations across all diagnosis groups dropped substantially below baseline levels (Figure 4:  most impacted diagnoses; Supplementary Figure 12, Table 3: all diagnoses). Intestinal infections and non–COVID-19 respiratory illnesses experienced the most pronounced and sustained declines, with some diagnoses reduced below 80% of baseline, even as NPIs were relaxed (Figure 4, Supplementary Table 3). Admissions for injuries and noncommunicable diseases were also affected, declining sharply during home confinement and respectively increasing up to 20% and 10%–40% below baseline during more relaxed public health measures (Figure 4, Supplementary Figure 12, Table 3). By September 2021, total admissions were approximately 30% below baseline, with respiratory illnesses and intestinal infections ≥35% and noncommunicable diseases ≥20% below baseline (Supplementary Table 3).

Similar to trends observed for hospitalizations, outpatient visits for intestinal infections and respiratory illnesses, followed by injuries, were the most impacted by the pandemic and had not recovered to pre-pandemic levels by the end of the study (Supplementary Figures 13 and 14, Tables 4 and 5). ED attendances experienced greater overall declines in patient volume than GP consultations. The percentage of telehealth appointments out of total GP consultations was the lowest in March 2020 (0.4%) and the highest during the first COVID-19 wave in July 2020 (12.2%; Supplementary Figure 1). GP consultations for asthma, human immunodeficiency virus (HIV), and chronic illnesses were not substantially affected by lockdown restrictions and continued at baseline levels throughout much of the pandemic (Supplementary Figure 14). By September 2021, ED attendances remained depressed across most diagnoses, while GP consultations for HIV, noncommunicable diseases, and injuries were closer to projected baselines (Supplementary Tables 4 and 5).

National trends in non–COVID-19 consultations are described in detail in the Supplementary Materials.

### Associations Between Hospitalizations and COVID-19 Policies and Human Mobility

**Figure 5.**
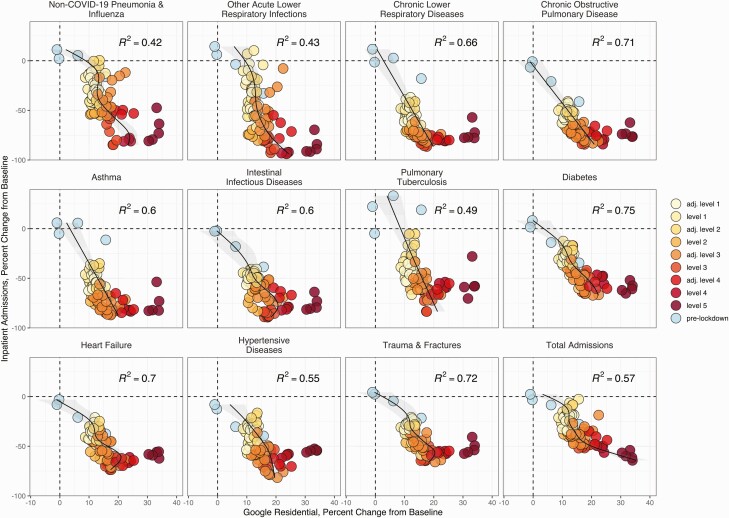
Visits to residential locations are associated with reduced admissions for non- COVID-19 conditions. Relationships between the weekly percent change from baseline in the Google Residential metric and the weekly percent change from baseline in total admissions and non–COVID-19 admissions. Point colors indicate the pre-lockdown period (1 March 2020–26 March 2020) and lockdown alert levels from March 2020 to September 2021 (Table 1). Points are shaded according to the stringency of lockdown measures: dark to light. Generalized additive models (GAMs) were used to identify nonlinear relationships between the Google Residential metric and inpatient admissions for each diagnosis group. GAM adjusted R^2^ values are in the top right of each facet. See Supplementary Figure 15 for plots of all diagnosis groupings analyzed in the study. Abbreviation: COVID-19, coronavirus disease 2019.

Admissions for chronic respiratory diseases (eg, asthma) and nonrespiratory conditions (eg, intestinal infections, diabetes, injuries) were strongly associated with mobility indicators and NPI strength (visits to residential locations: Figure 5, Supplementary Figure 15; visits to transit stations: Supplementary Figure 16). Mobility indicators preceded or coincided with declines in admissions, but temporal lags between NPIs and admissions for some conditions changed over the course of the pandemic (Supplementary Figure 17). For example, declines in admissions for chronic and acute illnesses, intestinal infections, and injuries occurred synchronously with NPIs during the first pandemic wave in July 2020, while they preceded NPIs during the second wave. The most pronounced reductions in mobility and injuries coincided with alcohol bans (Supplementary Figure 18, Table 1).

In contrast to chronic conditions and infectious diseases unrelated to COVID-19, hospitalizations for all-cause respiratory conditions and, specifically, COVID-19 were not linearly associated with mobility or policy stringency (Supplementary Figure 19), and these indicators were not generally predictive of COVID-19 burden (Supplementary Figure 20).

## DISCUSSION

We utilized counterfactual time series analyses to assess the direct and indirect impacts of the COVID-19 pandemic on private healthcare utilization in South Africa from March 2020 to September 2021. Outpatient respiratory visits spiked in mid-March 2020, consistent with “worried-well” behavior. The nationwide lockdown 2 weeks later precipitated pronounced declines across all diagnosis groups at all levels of severity. The first wave of all-cause respiratory encounters followed the initial relaxation of stay-at-home orders in May 2020, and the latter 2 waves were linked to novel, highly transmissible viral variants. Despite the relatively smaller waves of GP consultations, excess all-cause respiratory encounters correlated strongly with COVID-19–coded encounters across all consultation types, demonstrating the utility of syndrome-based analyses for monitoring pandemics. Respiratory and specifically COVID-19–coded hospitalizations did not generally align with NPIs or patterns in human mobility. In contrast, nonrespiratory admissions remained substantially below baseline levels after March 2020 and exhibited strong linear relationships with both public health measures and trends in population-level mobility.

Hospitalizations and ED visits for acute medical events (eg, heart attacks, strokes) and chronic illnesses (eg, diabetes, hypertension) were below baseline throughout the pandemic, despite the lifting of shelter-in-place orders beginning in May 2020. Many of these currently below-baseline conditions are risk factors for or complications of severe COVID-19 disease and associated with excess mortality [19–21]. Declines could arise from patients avoiding or delaying seeking care due to stay-at-home restrictions, fear of COVID-19, loss of employer-sponsored health insurance, better self-management of symptoms, genuine reductions in incidence (eg, declines in injuries due to fewer traffic accidents and alcohol-related incidents [11, 22]), increased thresholds for hospitalization, and the cancellation of elective procedures during each COVID-19 surge [23]. The monthly percentage of telehealth visits did not exceed 10%, to during South Africa’s first COVID-19 peak. Thus, the displacement of in-person care to telemedicine accounted for a small proportion of observed reductions in physical primary care appointments. Decreased diagnoses for acute and chronic conditions may translate into morbidity and mortality increases in the future if patients avoided or were turned away from the system. For example, England and Wales experienced an abrupt increase in acute cardiovascular deaths during March 2020–June 2020, with 50% occurring outside of hospital settings [20]. South Africa’s detailed mortality statistics have a 3-year lag; thus, the downstream effects of delayed care, both short-term and long-term, may not be quantifiable for several years.

Notably, physical distancing and school closures significantly reduced the transmission of seasonal respiratory viruses in South Africa [24], as observed in other countries [24–26]. Widespread mitigation measures for COVID-19 delayed South Africa’s RSV season [27], effectively eliminated influenza circulation [24, 27], and likely prevented outbreaks of diarrheal diseases [28]. Accordingly, encounters for non–COVID-19 respiratory illnesses and intestinal infections decreased at all levels of severity. In addition to NPIs, changes in routine healthcare-seeking might explain reductions in mild cases of respiratory illness or gastroenteritis. However, the declines observed for severe cases, especially in young children, indicate an overall beneficial impact of social distancing, school closures, and increased hygiene measures on transmission of endemic pathogens [26, 28].

In children, all-cause respiratory encounters spiked in late February 2021, consistent with a resurgence of RSV in the last quarter of 2020. February is typically the start of the RSV season in South Africa, and this epidemic was smaller than in past seasons [27, 29]. Though influenza activity has remained low in South Africa, continuing influenza surveillance and promoting vaccination is essential [24, 27], as the buildup of susceptible individuals could lead to larger epidemics in the future [30].

Many studies have linked COVID-19 dynamics to variation in NPIs and mobility behavior [31–33], particularly during the early phases of the pandemic. Public health measures were inversely correlated with all-cause respiratory hospitalizations and, specifically, COVID-19 hospitalizations during the few weeks preceding and including each COVID-19 peak but did not track with smaller-scale fluctuations in weekly hospitalizations. After the initial relaxation of lockdown restrictions in May 2020, respiratory admissions decoupled from NPIs and mobility patterns. This lack of concordance suggests that mobility metrics and NPI stringency may not fully capture the behavioral drivers of SARS-CoV-2 transmission and associated hospital surges due to “pandemic-policy fatigue.” Further, increasing population immunity may contribute to case declines to a greater extent than mobility reductions in later phases of the pandemic.

A strength of our study is its large, comprehensive dataset encompassing almost 150 providers across all 9 provinces and the 6 years leading up to and including the pandemic, which is uncommon for African countries. The majority of studies that assessed the effects of COVID-19 on healthcare utilization have focused on high-income countries and were limited in scope to specific age groups and medical conditions. Here, we gain broader insight into the pandemic’s impact on epidemiology by analyzing medical encounters across diagnoses and age groups and at all levels of the severity pyramid.

A major limitation is that our data are derived from 1 private healthcare group and may not be representative of the private sector as a whole or generalizable to the public sector. Aside from age, we did not have demographic data for Netcare’s patient population. However, patients with private health insurance are known to have greater access to healthcare, are more inclined to seek care [34], and tend to be white, high income, and live in urban and suburban areas [35] where private hospitals and doctors are concentrated [34]. The public sector has a higher burden of chronic diseases, HIV, and tuberculosis because of these disparities [21], and public sector patients were less likely to receive diagnosis and treatment for any condition when health systems were overwhelmed by the pandemic [36]. Analyzing trends in the public sector would provide a more complete view of healthcare use, but comparable data may not be available in the short term [37]. Second, due to data limitations, we could not assess the pandemic’s impact on medication access or whether delayed care was associated with elevated community or in-hospital mortality rates. Last, our national study could obscure geographic heterogeneities.

## CONCLUSION

The private health sector in South Africa experienced marked, persistent declines across diagnosis groups and consultation types during the pandemic. Strategies to reduce SARS-CoV-2 transmission and strain on the health system had a beneficial impact on the incidence of other respiratory illnesses, intestinal infections, and injuries. However, admissions and ED visits for many acute and chronic illnesses had not recovered to pre-pandemic levels by the end of the study, indicating that high-risk patients are still avoiding or deferring seeking care. Understanding the direct and indirect effects of the pandemic can inform the provision of health services and public health messaging as countries return to normalcy or if stricter interventions need to be implemented again [3]. Medical providers and public health agencies should prioritize maintaining access to health services and encourage routine checkups and seeking immediate treatment for serious illnesses.

## Supplementary Data

Supplementary materials are available at *Clinical Infectious Diseases* online. Consisting of data provided by the authors to benefit the reader, the posted materials are not copyedited and are the sole responsibility of the authors, so questions or comments should be addressed to the corresponding author.

ciac055_suppl_Supplementary_Material

ciac055_suppl_Supplementary_Material_1

## Notes

***Author Contributions.*** A. C. P., S. T., C. V., and C. C. conceived of and designed the study, with contributions from J. B. and J. R. C. P.; C. M., M. T., A. L., S. W., and C. C. provided the data; and S. T. and S. W. curated the data. A. C. P. and C. V. developed the methodology, with inputs from S. T., J. B., J. R. C. P., and C. C. In addition, A. C. P. performed the formal analysis, created the figures, and wrote the original draft of the manuscript. All coauthors reviewed and edited the draft and gave final approval for publication.

***Acknowledgments*.** The authors thank all members involved in the pneumonia and influenza-like illness surveillance programs for the collection and processing of specimens and the data management team at the National Institute for Communicable Diseases for data quality control and assurance.

***Disclaimer.*** The funders had no role in the design and conduct of the study; collection, management, analysis, and interpretation of the data; preparation, review, and approval of the manuscript; or the decision to submit the manuscript for publication. The conclusions of this study do not necessarily represent the views of the National Institutes of Health or the US government. Any opinion, finding, conclusion or recommendation expressed in this material is that of the authors; the National Research Foundation does not accept any liability in this regard.

***Financial support.*** This work was supported by the in-house research division of the Fogarty International Center, US National Institutes of Health (A. C. P. and C. V.), the Department of Science and Innovation, South Africa (J. R. C. P. and J. B.), the National Research Foundation, South Africa (J. R. C. P. and J. B.), the National Institute for Communicable Diseases of the National Health Laboratory Service, South Africa (S. T., S. W., and C. C.), and the Wellcome Trust and United Kingdom Foreign, Commonwealth and Development Office (grant 221003/Z/20/Z to C. C., C. V., and J. R. C. P.).

***Potential conflicts of interest.*** M. T. reports leadership in the Netcare Foundation, Emergency Medicine Society, and the Netcare Emergency Transplant Trauma corporate social investment (CSI). J. R. C. P. reports grants from the Wellcome Trust, Bill & Melinda Gates Foundation, National Research Foundation (South Africa), and US National Science Foundation outside the submitted work; contracts from the World Health Organization Regional Office for Africa (WHO AFRO), South African National Blood Service, and the Tuberculosis (TB) Think Tank via the Aurum Institute, South Africa; consulting fees from Africa Risk Capacity; and advisory board membership for the Prospective Household Study of SARS-CoV-2, Influenza, and Respiratory Syncytial Virus Community Burden, Transmission Dynamics, and Viral Interaction in South Africa (PHIRST-C) study, the National Institute for Health Research (NIHR) Global Health Research Group on the Application of Genomics and Modelling to the Control of Virus Pathogens (GeMVi) in East Africa, and the Ministerial Advisory Committee on COVID-19 (coronavirus disease 2019), National Department of Health, South Africa; support for attending meetings and/or travel from Public Health England and National Advisory Council on Innovation (South Africa); serving as director, International Clinics on Infectious Disease Dynamics and Data Programme and member, Ministerial Advisory Committee on COVID-19, National Department of Health, South Africa; and serving on editorial boards for Epidemics (Elsevier), Proceedings of the Royal Society B, Philosophical Transactions of the Royal Society, and Scientific Committee, Epidemics 8 (Elsevier-run conference). C. C. reports grants from the Wellcome Trust, South Africa Medical Research Council, US Centers for Disease Control and Prevention, and Sanofi Pasteur outside the submitted work and advisory board membership for the Burden of COVID-19 among Healthcare Workers Study. All other authors report no potential conflicts.

***Data availability.*** Publicly available data on national COVID-19 testing, mobility behavior, and COVID-19 policies were obtained from Our World in Data, Google Community Mobility Reports, and the Oxford COVID-19 Government Response Tracker. Data from pneumonia and influenza-like illness surveillance programs were provided directly to the authors by the National Institute for Communicable Diseases. Aggregated data on code-specific primary care visits, emergency department presentations, and hospital admissions were provided directly to the authors by Netcare. The investigators welcome enquiries about possible collaborations and requests for access to datasets. Data will be shared after approval of a proposal and with a signed data access agreement. Investigators interested in accessing these resources should contact the corresponding author. R code for time series analyses and figures can be accessed in GitHub (https://github.com/aperofsky/South_Africa_Healthcare_Use_during_COVID).
