# Supplementary material for: The direct and indirect effects of the COVID-19 pandemic on private healthcare utilization in South Africa, March 2020 – September 2021
Source: Clin Infect Dis. 2022 Jan 27:ciac055. doi: 10.1093/cid/ciac055 (PMC8807275; doi:10.1093/cid/ciac055)
Supplement: Supplementary file 1 [file ciac055_suppl_supplementary_material.pdf]

Supplementary Material for:

Perofsky et al. "The direct and indirect effects of the COVID-19 pandemic on private healthcare utilization in South Africa, March 2020 – September 2021"

Supplementary Methods

Descriptions of respiratory illness surveillance programmes in South Africa

The ILI surveillance programme systematically collect samples from patients who present with acute respiratory illness, fever (> 38°C), and cough at public clinics in KwaZulu-Natal, Western Cape, North-West, and Mpumalanga provinces. The pneumonia surveillance programme collects samples from hospitalized patients with severe respiratory illness, at sites located in KwaZulu-Natal, Mpumalanga, North-West, Gauteng, and Western Cape provinces. Respiratory samples are tested at NICD for influenza, RSV, and SARS-CoV-2 (see [1,2]).

Human mobility and government responses to COVID-19 in South Africa

Google provides aggregated and anonymized cell phone location data from users with a Google account on their cell phone who opted to have their location data available to Google Location History. We obtained national-level mobility trends for South Africa in six location categories: retail and recreation, grocery and pharmacy, parks, residential, transit stations, and workplaces (Figure 1) [3]. For each category, Google reports the daily percent change from baseline activity, with the baseline defined as the median value for that day of the week between 3 January and 6 February 2020.

During the strictest phase of the lockdown (Level 5), visits to workplaces, transit stations, and retail and recreation locations were the most impacted mobility categories, with declines of up to 70% below baseline activity (Figure 1). Visits to parks and groceries and pharmacies dropped to approximately 50% below baseline levels, while visits to residential locations increased to levels greater than 25% above baseline activity. Mobility metrics for non-residential locations increased towards baseline levels following the initial easing of shelter-in-place orders in May 2020 (Level 4). After the transition to adjusted Level 3 in December 2020, visits to non-residential locations sharply declined but then increased, with some categories reaching (workplaces, retail and recreation) or exceeding (grocery and pharmacy) pre-pandemic levels by late May 2021 (adjusted Level 2). Visits to non-residential locations dipped again after the move to adjusted Level 4 in late June 2021 but rebounded after the initiation of adjusted Level 3 in late July 2021. Throughout the pandemic, visits to groceries and pharmacies were the least impacted by policy measures because these businesses were categorized as essential services.

We extracted data collected by the Oxford COVID-19 Government Response Tracker (OxCGRT)[4] to measure variation in South African government policies to COVID-19 from March 2020 to September 2021. The OxCGRT database tracks publicly available information for policies related to closure and containment, health, and economic policy in 180 countries, recording policy responses on ordinal or continuous scales for 19 policy areas. We obtained daily values for the stringency index, which combines all containment and closure (C) indicators and the H1 indicator (public information campaigns) (Figure S6, Table S1).

We averaged daily values for Google Mobility categories and the Oxford Stringency Index by epidemic week to match the resolution of medical encounter data.

Cross-correlations between hospitalizations and the Google Residential metric or the Oxford Stringency Index

For each diagnosis group, we computed time series cross-correlations between the percent change from baseline in inpatient admissions and the percent change from baseline in the Google Residential metric (GRM) or the Oxford Stringency Index (OSI) in rolling 20-week windows. For each 20-week window, we extracted the maximum coefficient value and the lag (in weeks) at which this value occurred (the 'optimal lag'). Negative lag values indicate the GRM or OSI time series leads the admissions time series, and positive lag values indicate the GRM or OSI time series lags behind admissions. A lag of 0 indicates that two time series are in phase. To test whether cross-correlation coefficients were significantly higher or lower than expected by chance, we used permutation tests to generate null distributions of cross-correlations. At each 20-week time window, we generated 1000 block bootstrap samples of the GRM or OSI time series with three-week block lengths (*tseries* package)[5] and recomputed cross-correlations between hospitalizations and GRM or OSI for each replicate, yielding a null distribution of 1000 cross-correlations. Cross-correlations between admissions and GRM or OSI were considered statistically significant when observed coefficients were outside the bounds of the null distribution's 95% confidence interval.

Supplementary Results and Discussion

Age-specific patterns in all-cause respiratory admissions

In individuals aged ≥ 5 years, there were 55,927 all-cause respiratory hospitalizations from March 2020 to March 2021, compared with an annual average of 43,197 (95% confidence interval (CI): [36,942, 49,453]) during the same period in the previous four years (Figure 3). In individuals aged < 5 years, there were 6,152 all-cause respiratory hospitalizations from March 2020 to March 2021, compared with an annual average of 19,948 [17,506, 22,391] during the same period in the previous four years (Figure 3).

Since the initiation of lockdown measures on 26 March 2020, respiratory hospitalizations in young children (< 5 years) have been substantially below those expected in the absence of COVID-19 ($\bar{x}$ = 40% [27, 52] below baseline, Figure 3), consistent with diminished transmission of RSV, influenza, and other seasonal viruses [6] (Figure 2). Respiratory hospitalizations in young children increased in November 2020, reflecting the increasing circulation of RSV (Figure 3). Admissions in older children (5-19 years) gradually increased after the easing of lockdown measures in May 2020, suggesting greater exposure to SARS-CoV-2 or other respiratory pathogens outside the home. All-cause respiratory admissions in adults increased after the initiation of lockdown measures and, in early July 2020, reached levels several times higher than those observed in past influenza seasons (peak increase above baseline: 20-49 years, 230% [154, 342]; ≥ 50 years, 275% [185, 406]). After the first COVID-19 wave, hospitalizations in adults remained slightly above the seasonal baseline from mid-August to November 2020 before rapidly increasing during November and December 2020 to levels higher than those reached during the first wave (peak increase above baseline: 20-49 years, 347% [224, 527]; ≥ 50 years, 464% [310, 689]). In children, all-cause respiratory admissions spiked in late February 2021 (peak increase above baseline: < 5 years, 153% [95, 298]; 5-19 years, 74% [14, 205]), consistent with increasing RSV circulation. Inpatient admissions in children declined to below seasonal baseline levels after the transition to adjusted Level 1 on 1 March 2021. After the second COVID-19 wave culminated in late January 2021, adult hospitalizations continued at levels equivalent to the seasonal baseline until the Delta variant sparked a third wave of infections in June 2021. In late June/early July 2021, hospitalizations for both adult age groups peaked at levels similar to numbers observed during the second wave (peak increase above baseline: 20-49 years, 233% [140, 396]; ≥ 50 years, 433% [278, 635]). The weekly percent change in baseline for all-cause respiratory admissions strongly correlated with weekly COVID-19-coded admissions in adults (20-49 years: $R^2 = 0.93$, $P < 0.001$; ≥50 years: $R^2 = 0.97$, $P < 0.001$) but not in children (Figure S11).

National and age-specific trends in outpatient all-cause respiratory encounters

In individuals aged ≥ 5 years, there were 36,692 all-cause respiratory attendances at emergency departments (ED) in private hospitals and 229,723 consultations at private general practitioners from March 2020 to March 2021, compared with averages of 39,265 [36,917, 41,613] and 391,056 [318,287, 463,827], respectively, during the same period in the previous four years (Figures S8-9). In individuals aged < 5 years, there were 6,992 all-cause respiratory ED visits and 57,870 GP visits compared to annual averages of 23,066 [20,575, 25,556] and 518,148 [423,429 , 612,866], respectively (Figures S9-10).

Outpatient all-cause respiratory consultations spiked nationally and across all age groups during the week of 15 March 2020, consistent with "worried well" health-seeking behavior (national peak increase above baseline: ED, 279% [150, 459]; GP, 150% [108, 214]; see Figure 2 and Figure S8 for national trends and Figures S9-10 for age-specific trends). After the onset of Level 5, outpatient respiratory visits declined sharply across all ages, reflecting the impact of lockdown measures. Adult ED and GP respiratory encounters increased after the transition to Level 3 in June 2020, peaked at levels above (ED) or equivalent to (GP) their seasonal baselines in July 2020, and then declined below baseline levels in August 2020 (Figures S9-10). Like trends observed in inpatient admissions, national-level and adult ED respiratory visits during the second wave in December 2020 exceeded levels observed during the first wave (national peak increase above baseline: 159% [70, 242]; Figure 2; Figures S8-9). GP respiratory consultations also peaked in late December 2020, with consultations in adults aged ≥ 20 years surpassing baseline levels (Figure 2, Figure S8, Figure S10). The third wave of outpatient visits peaked in early July 2021, with national ED visits peaking at a level equivalent to the projected baseline (12% [-24, 87]) and adult visits exceeding baseline levels (Figure S9). National and adult GP visits surpassed those observed during the prior two waves (peak increase above baseline: 60% [26, 107]; Figure S8, Figure S10).

After the initiation of lockdown Level 5 in late March 2020, outpatient respiratory visits in children (< 5 years, 5-19 years) remained substantially below baseline numbers until RSV circulation increased in last quarter of 2020 (Figures S9-10). ED and GP consultations in young children spiked in February 2021 and then declined below their

seasonal baselines. During the third COVID-19 wave, GP visits in individuals aged < 20 years increased to baseline levels, whereas ED visits appeared unaffected.

Like trends observed in inpatient admissions, the weekly percent change in baseline for outpatient all-cause respiratory consultations strongly correlated with weekly COVID-19-coded admissions in adults aged ≥ 20 years but not in children (Figure S11).

Non-COVID-19 hospital admissions

From March 2020 to March 2021, there were 370,521 total admissions and 302,131 non-respiratory admissions, compared to an average of 598,094 [577,429, 618,759] annual total admissions and 516,080 [503,480, 528,679] annual non-respiratory admissions during the same period in the previous four years.

From 1 March 2020 to the lockdown on 26 March 2020, weekly inpatient admissions were equivalent to or slightly above their projected baselines (Figure S12, Table 1). During the strictest lockdown phases (Levels 5 to 4), hospitalizations across all diagnosis groups dropped substantially below baseline levels. Intestinal infections and non-COVID respiratory illnesses experienced the most pronounced and sustained declines, with some diagnoses reduced below 80% of baseline, even as NPIs were relaxed (Table 1). During less stringent NPIs (April - May 2021), admissions for acute respiratory infections rebounded to 20% below baseline, while those for chronic lower respiratory illnesses, specifically asthma and COPD, continued at levels 50% below baseline. Admissions for injuries and non-communicable diseases were also impacted, declining sharply during home confinement and respectively increasing up to 20% and 10-40% below baseline during more relaxed public health measures (Figure S12, Table 1). At the end of the study in September 2021, total admissions were 33.2% [29, 37.4] below baseline, non-COVID respiratory illnesses and intestinal infections were 35-65% below baseline, and non-communicable diseases were 20-40% below baseline. Cancer-related admissions were 33.6% [26.5, 40.7] below baseline at the end of the study, with year-to-date admittances for cancer surgeries and conventional radiation therapy 30% and 19% below numbers observed in 2019.

Non-COVID-19 consultations at emergency departments

From March 2020 to March 2021, there were 335,721 total attendances and 292,156 non-respiratory attendances at emergency departments, compared to an average of 552,725 [538,930, 566,521] annual total attendances and 490,395 [479,633, 501,157] annual non-respiratory attendances during the same period in the previous four years.

During the pre-lockdown period (1 – 26 March 2020), weekly attendances for non-COVID-19 respiratory illnesses (pneumonia and influenza, acute lower respiratory infections, chronic lower respiratory illnesses, and asthma) were at levels equivalent to or slightly above their projected baselines (Figure S13, Table S3). Attendances for intestinal infectious diseases, chronic diseases (COPD, diabetes, heart disease, heart failure, hypertension), neoplasms, alcohol-related disorders, and injuries were also equivalent to baseline levels. After the initiation of strict lockdown measures (Level 5), attendances for respiratory illnesses, intestinal infectious diseases, heart diseases, cerebrovascular diseases, alcohol-related disorders, and injuries declined to levels 50% below baseline, while attendances for other conditions, such as acute myocardial infarctions (AMI), heart failure, and hypertension, exhibited less marked declines (Figure S13, Table S3). Acute and chronic respiratory illnesses, COPD, intestinal infectious diseases, and injuries experienced the most pronounced declines throughout the pandemic, while attendances for non-communicable diseases returned to numbers closer to baseline levels during periods of more relaxed public health measures. Though attendances for injuries increased during Levels 2 and 1 (August – mid-December 2020), they dropped again to numbers 50% below baseline when stay-at-home restrictions tightened in late December 2020. At the end of the study period in September 2021, attendances for non-COVID respiratory illnesses, intestinal infections, and alcohol-related incidents were approximately 50-75% below projected baselines, visits for heart diseases, heart failure, cerebrovascular diseases, and hypertension were 10-25% below baseline, and injuries were 35-45% below baseline (Figure S13, Table S3). In September 2021, encounters for diabetes and AMI were close to pre-pandemic levels.

Non-COVID-19 consultations at general practitioner providers

In-person consultations at private general practitioner providers were less impacted by pandemic-related factors than hospitalizations and emergency department visits. From March 2020 to March 2021, there were 1,041,840 total consultations and 789,897 non-respiratory consultations at general practitioners, compared to an average of 2,084,934 [1,805,208, 2,364,660] annual total consultations and 1,480,505 [1,308,159, 1,652,851] annual non-respiratory consultations during the same period in the previous four years.

During the pre-lockdown period in March 2020, consultations for chronic lower respiratory illnesses, and in particular asthma, spiked at numbers 50% above seasonal baselines while consultations for other conditions were, on average, equivalent to baseline levels (Figure S14, Table S4). Non-COVID-19 pneumonia and influenza, acute lower respiratory infections, chronic bronchitis, and intestinal infectious diseases were the conditions most impacted by shelter-in-place orders, followed by injuries, neoplasms, and heart failure, and then chronic illnesses (e.g., lipidemias, heart diseases, and hypertension). Consultations for asthma, diabetes, and HIV were not, on average, impacted by the nationwide lockdown and remained at pre-pandemic levels throughout the pandemic. Similar to patterns observed for inpatient admissions and emergency department attendances, consultations for intestinal infectious diseases and some respiratory conditions, specifically pneumonia and influenza, acute lower respiratory infections, and chronic bronchitis, were 40-80% below baseline throughout the pandemic (Figure S14, Table S4). From May to September 2020 (Levels 4 to 2), consultations for diabetes, lipidemias, heart disease, heart failure, hypertension, HIV, and COPD were slightly below or equivalent to baseline levels, whereas those for neoplasms and injuries were approximately 20-25% below baseline. Weekly GP consultations for diabetes, lipidemias, heart disease, heart failure, hypertension, neoplasms, and injuries declined to numbers approximately 50% below baseline from late December 2020 to May 2021 (adjusted Levels 3 and 1) but returned to baseline levels in June 2021, after the transition to adjusted Level 2. At the end of the study in September 2021, GP visits for pneumonia and influenza, acute lower respiratory tract infections, chronic bronchitis, and intestinal infections were 50-60% below baseline, and those for chronic lower respiratory diseases, asthma, and COPD were 10-30% below baseline (Figure S14, Table S4). As of September 2021, consultations for neoplasms, cerebrovascular diseases, cardiac conditions, and injuries were 20-30% below baseline, while those for HIV, diabetes, and hypertension were close to pre-pandemic levels.

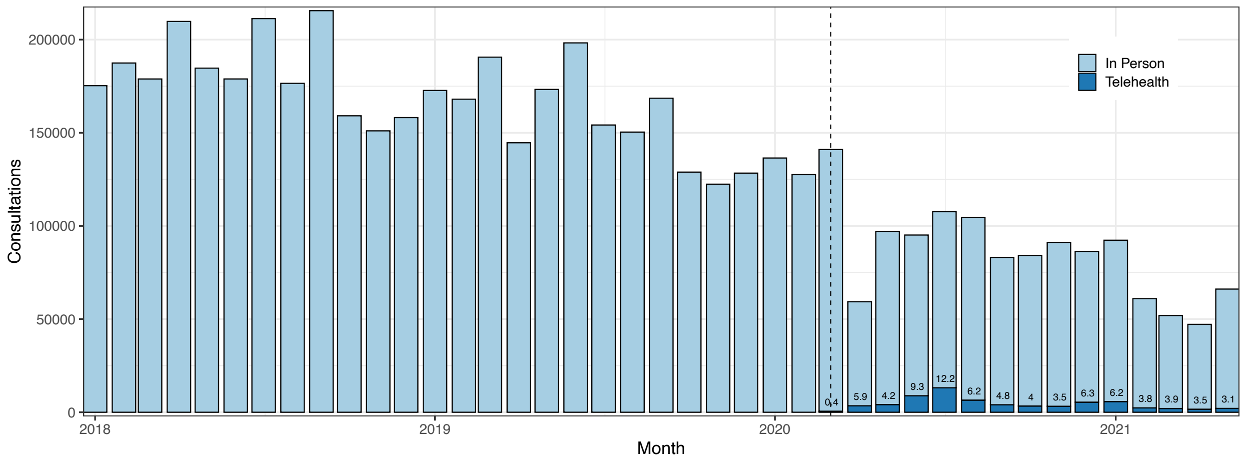

Figure S1. Numbers of monthly general practitioner consultations.
Medicross began its telehealth initiative in March 2020, indicated by the black vertical dashed line. From March 2020 to May 2021, visits are categorized as physical, in person visits (light blue) or as telehealth – telephone or virtual – consults (dark blue). Labels indicate the percentage of monthly GP visits that were telehealth consults.

The Health Professions Council of South Africa (HPSCA) regulates telemedicine in South Africa. Prior to the pandemic, HPSCA prohibited first-time consultations and required all telemedicine appointments to include a face-to-face consultation and physical examination by the 'consulting' practitioner in a clinical setting, with the 'consulting' practitioner communicating information to the 'servicing' practitioner (2014 Guidelines). The COVID-19 pandemic necessitated a relaxation of these policies to reduce healthcare worker contact with patients and to reach patients in remote and rural locations. The HPSCA amended their guidance in April 2020 to allow first-time consultations between clinicians and patients without an established relationship.

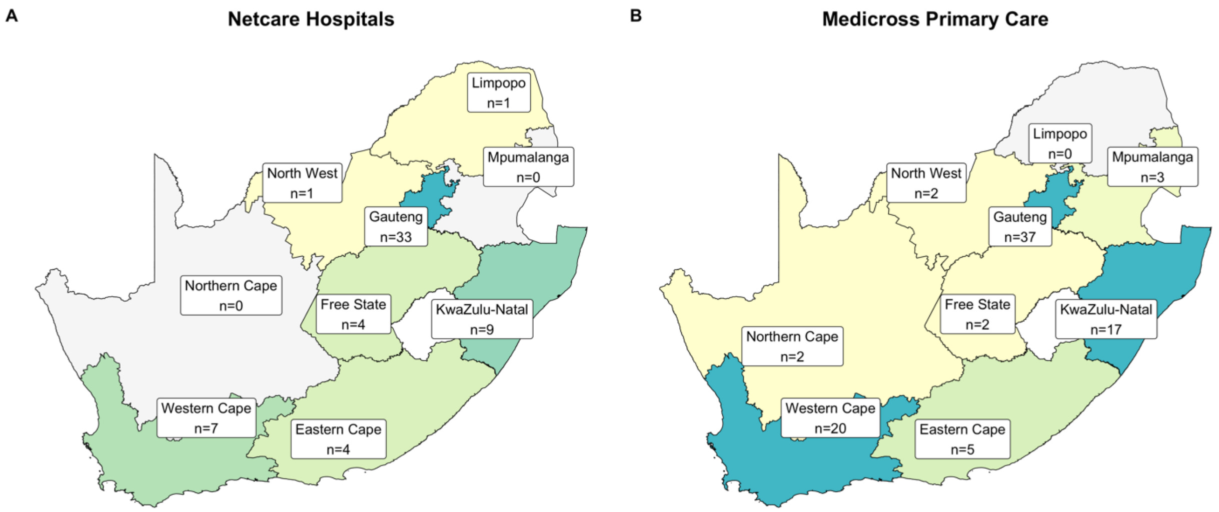

Figure S2. Maps of Netcare and Medicross provincial coverage.
Labels and shading indicate the number of **A.** Netcare hospitals and **B.** Medicross primary care clinics that reported medical encounters during the study period, with darker shading indicating higher numbers of reporting providers. Netcare and Medicross providers are concentrated in Gauteng, KwaZulu-Natal, and Western Cape.

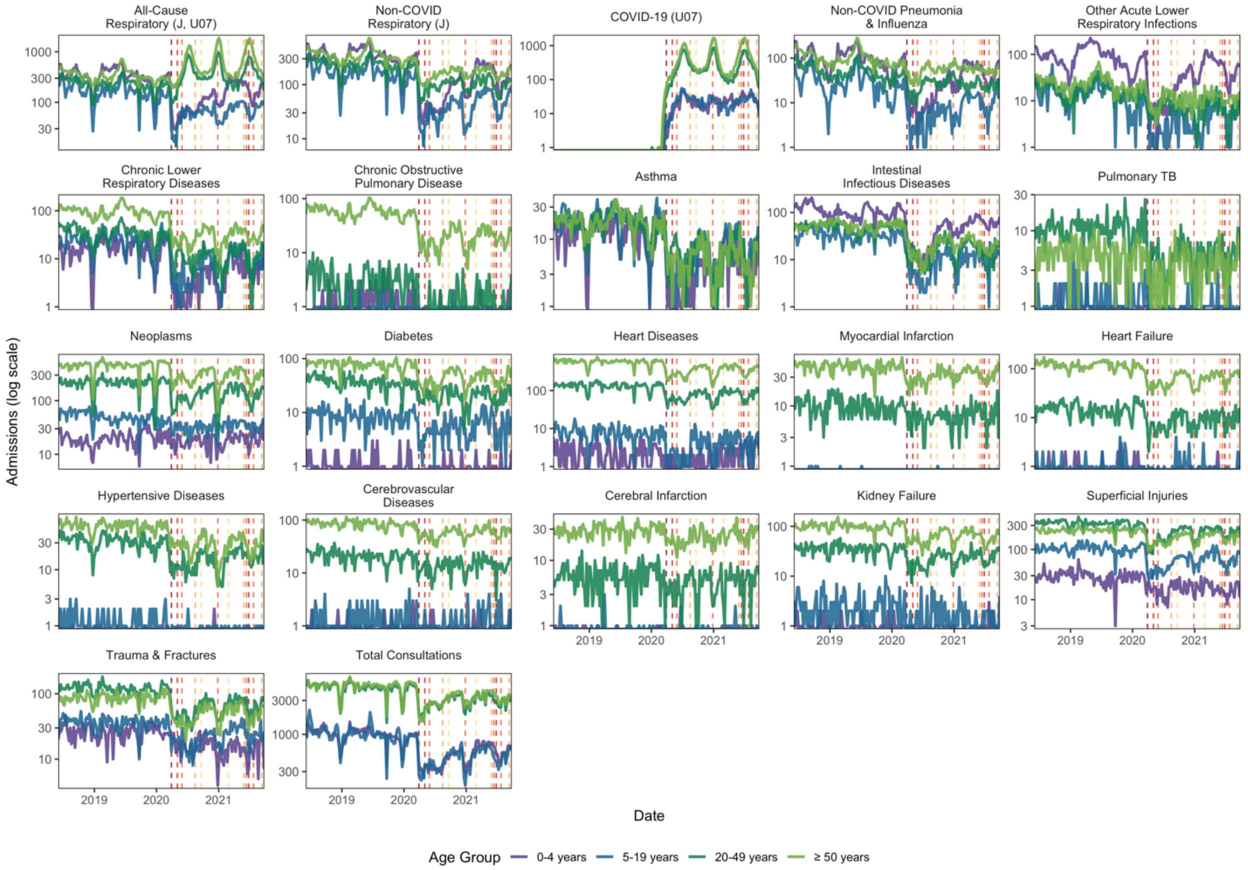

Figure S3. Weekly inpatient admissions by diagnosis and age group.
Vertical dashed lines indicate lockdown alert levels from March 2020 to September 2021 (Table 1) and are colored according to the stringency of lockdown measures: red to orange to yellow.

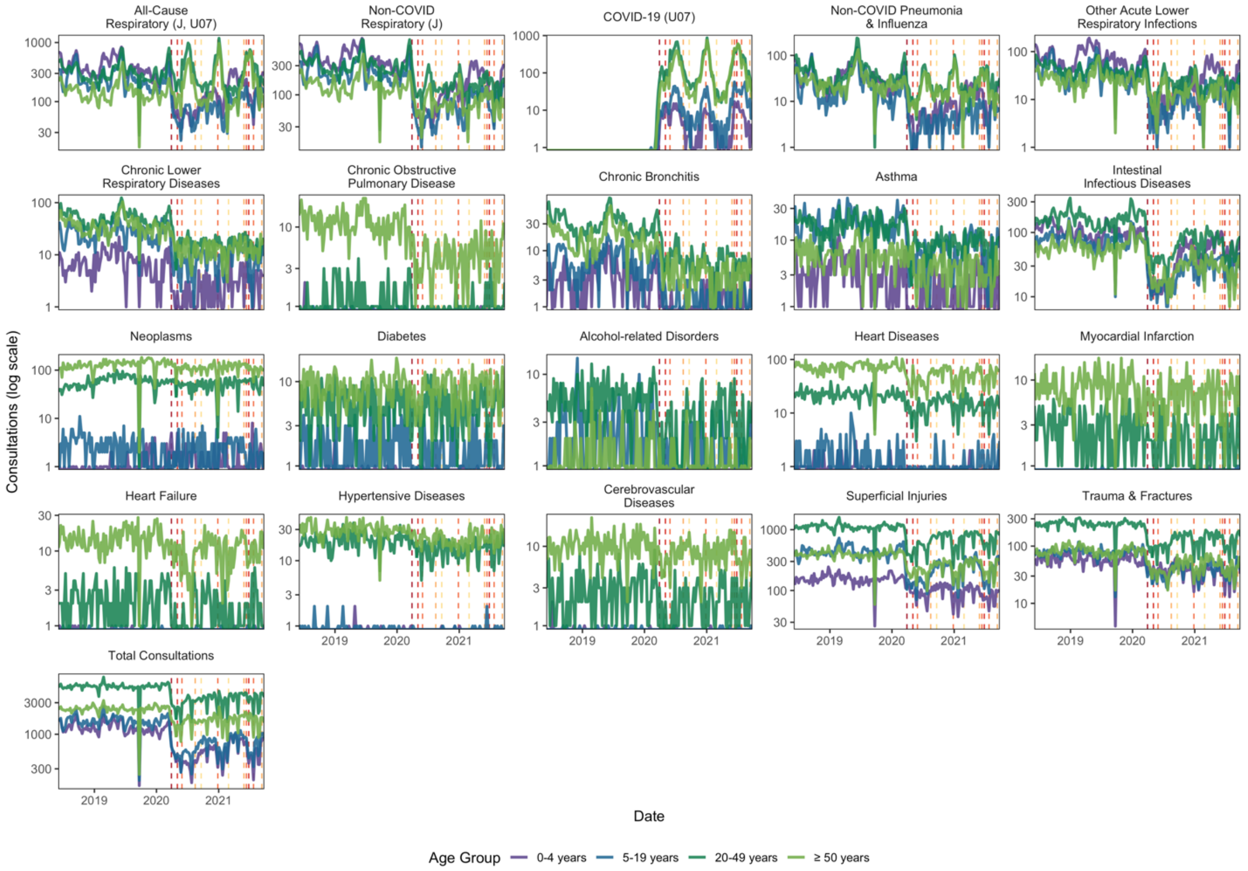

Figure S4. Weekly emergency department consultations by diagnosis and age group.
Vertical dashed lines indicate lockdown alert levels from March 2020 to September 2021 (Table 1) and are colored according to the stringency of lockdown measures: red to orange to yellow.

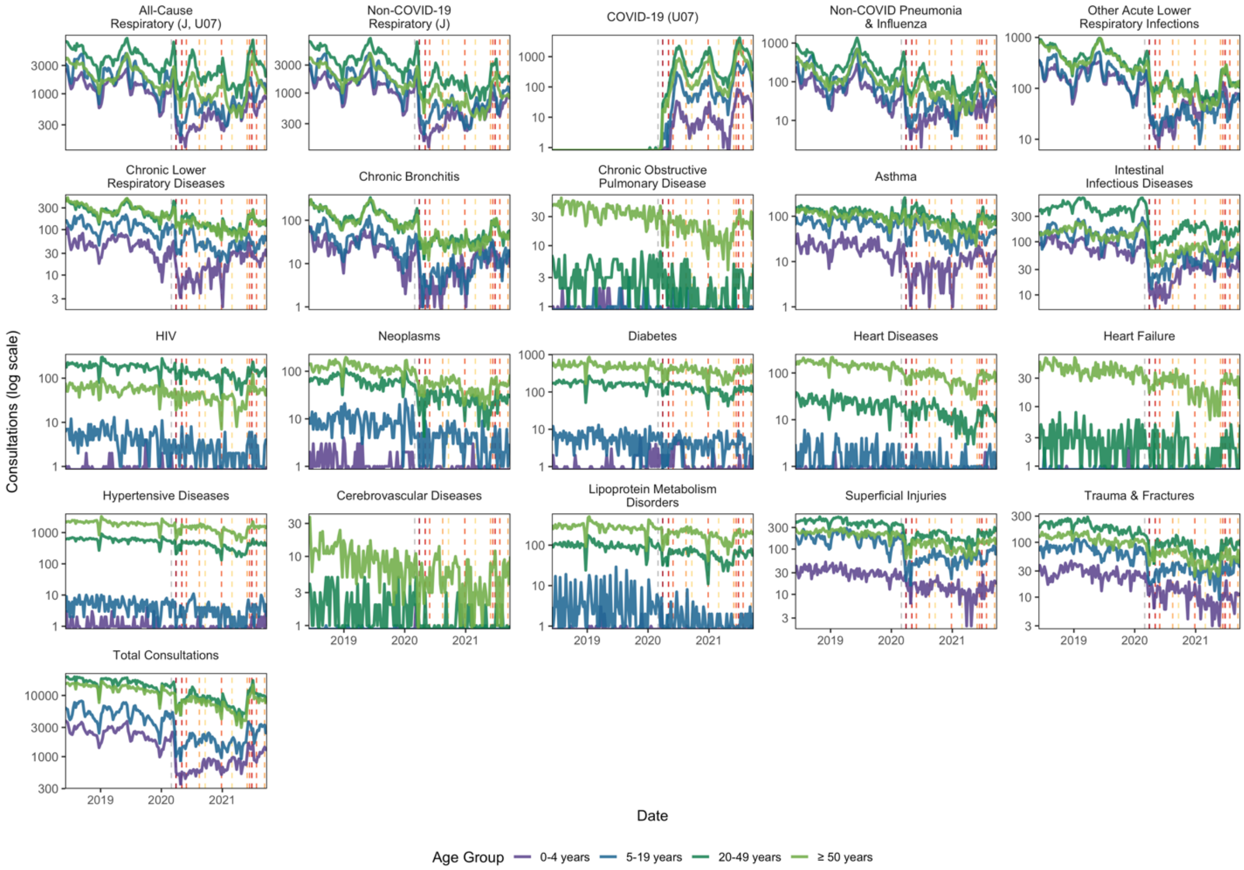

Figure S5. Weekly general practitioner consultations by diagnosis and age group.
Vertical dashed lines indicate lockdown alert levels from March 2020 to September 2021 (Table 1) and are colored according to the stringency of lockdown measures: red to orange to yellow.

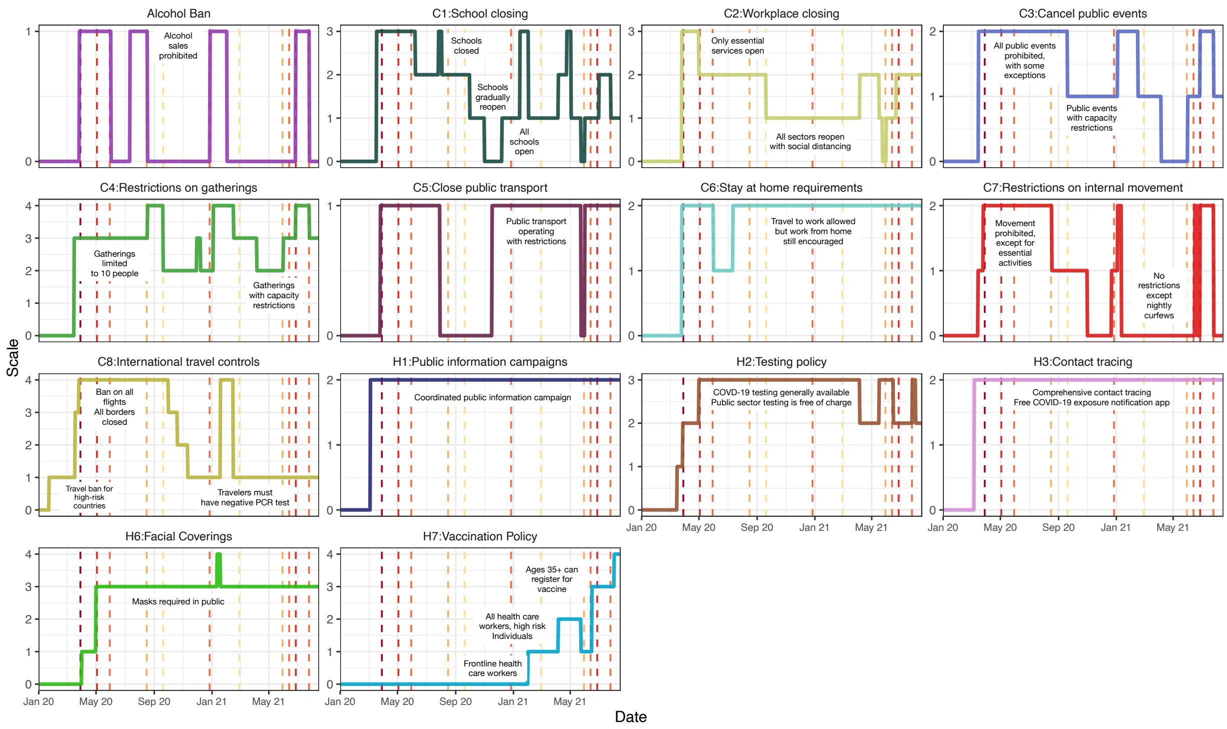

Figure S6. Individual indices for the ban of alcohol sales, containment and closure policies, and health policies in South Africa.

Daily time series for indicators C1 to C8, H1 to H3, and H6 to H7 were extracted from the OxCGRT database [4], which records individual indicators as ordinal or continuous values (Table S3). Zero values indicated no policy measures were taken. Vertical dashed lines indicate the stringency of lockdown measures: red to orange to yellow (Table 1). The policy stringency index combines indicators C1 to C8 and H1.

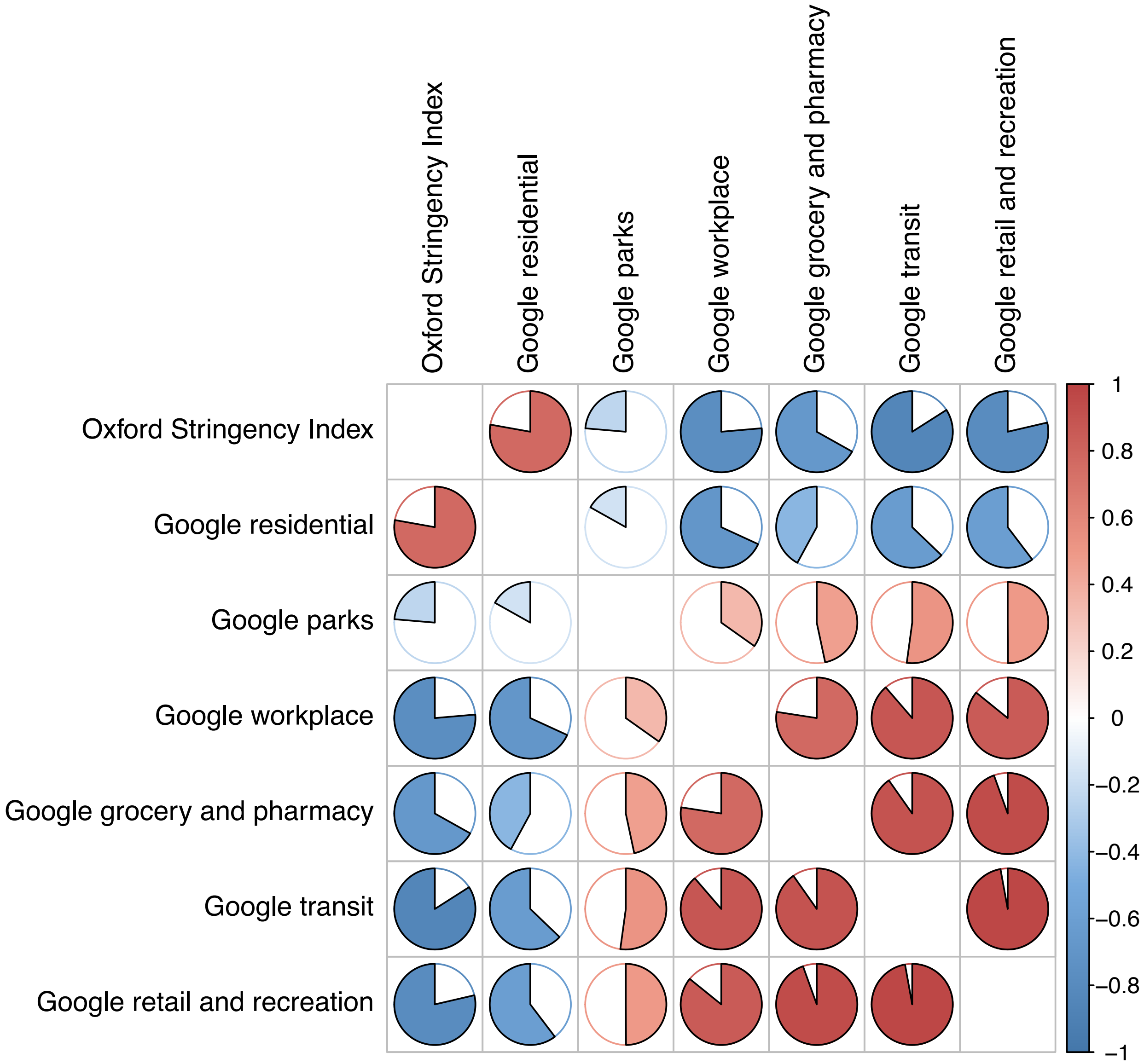

Figure S7. Pairwise Spearman correlations among the Oxford Stringency Index and Google Mobility metrics for South Africa.

Positive correlations are shaded in red, and negative correlations are shaded in blue. Color intensity and pie charts are proportional to correlation coefficients. Policy stringency was positively correlated with visits to residential locations and negatively correlated with visits to non-residential locations.

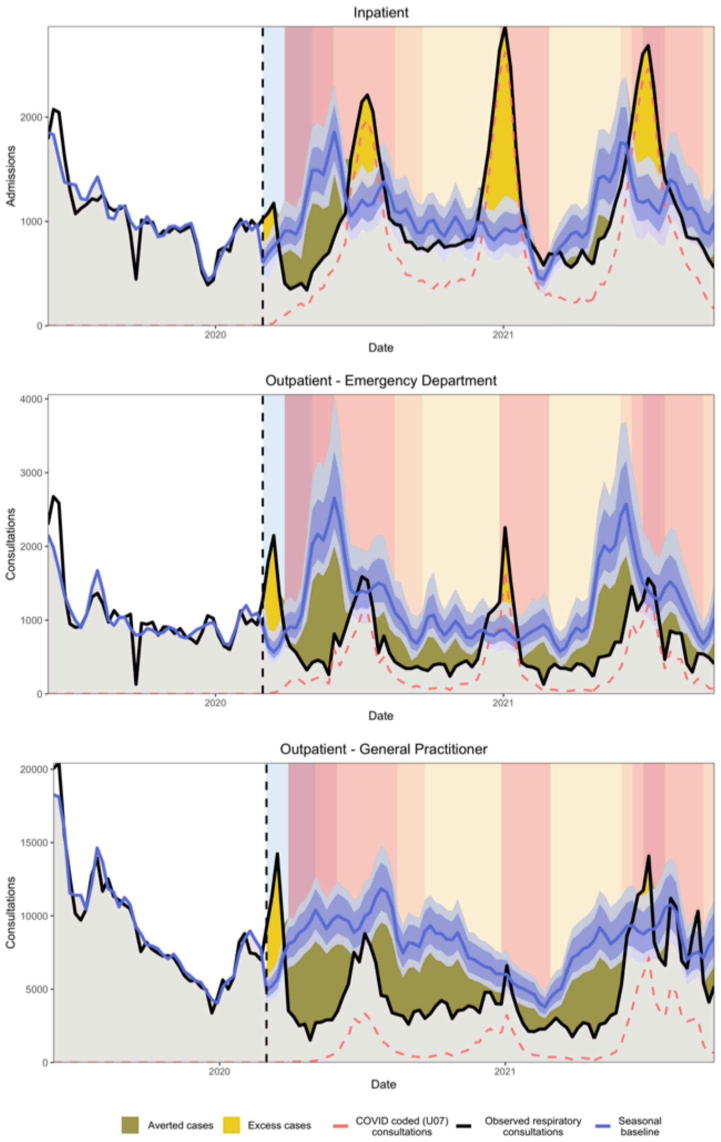

Figure S8. SARS-CoV-2 surveillance and all-cause respiratory encounters in individuals aged ≥ 5 years. Weekly all-cause respiratory consultations (including COVID-19) among individuals aged ≥ 5 years (black), relative to the baseline number of consultations expected in the absence of COVID-19 (blue band), at three levels of clinical severity: top: inpatient, middle. outpatient – emergency department, and bottom. outpatient – general practitioner. The blue band is the 95% prediction interval of the projected seasonal baseline. The red dashed line is the number of COVID-coded encounters. The black vertical dashed line indicates the start of the model prediction period (1 March 2020), and panel colors indicate the pre-lockdown period (light blue: 1 – 26 March 2020) and lockdown alert levels from March 2020 to September 2021 (Table 1). Panels are shaded according to the stringency of lockdown measures: red to orange to yellow. The area between the projected seasonal baseline and observed consultations is shaded green when observed consultations are below baseline ("averted cases") and shaded yellow when observed consultations are above baseline ("excess cases").

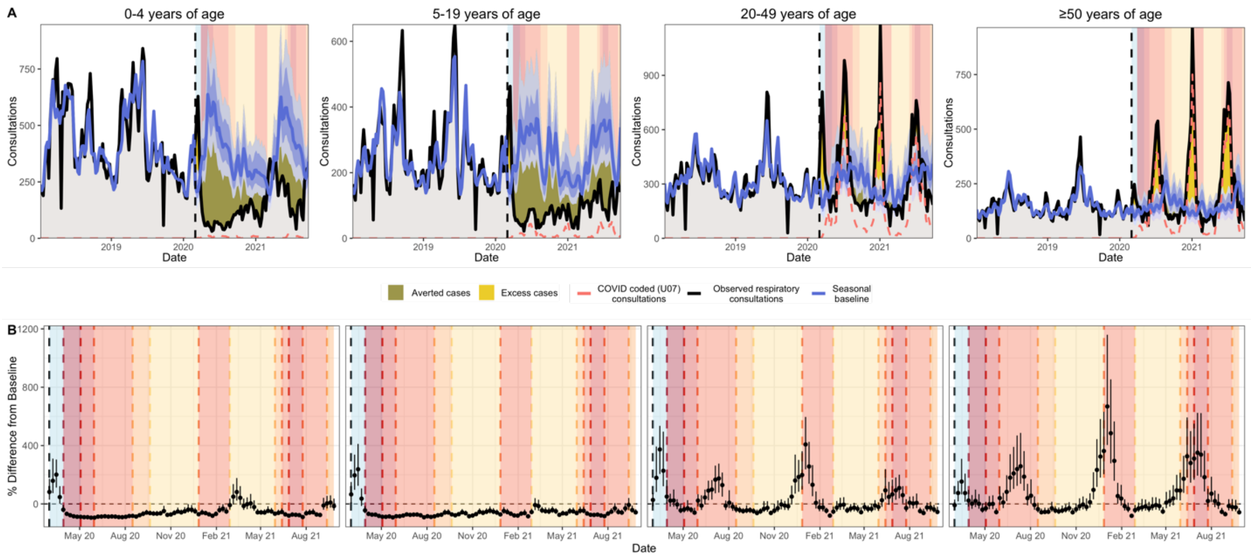

Figure S9. All-cause respiratory emergency department encounters in all age groups.
A. Weekly all-cause respiratory encounters by age group (black), relative to all-cause respiratory encounters expected in the absence of COVID-19 (blue band). The blue band is the 95% prediction interval of the projected seasonal baseline. The red dashed line is the number of COVID-coded encounters. The black vertical dashed line indicates the start of the model prediction period (1 March 2020), and panel colors indicate the pre-lockdown period (light blue: 1 – 26 March 2020) and lockdown alert levels from March 2020 to September 2021 (Table 1). Panels are shaded according to the stringency of lockdown measures: red to orange to yellow. The area between the projected seasonal baseline and observed consultations is shaded green when observed consultations are below baseline ("averted cases") and shaded yellow when observed consultations are above baseline ("excess cases"). **B.** Weekly observed percent difference from seasonal baseline (95% confidence interval) by age group.

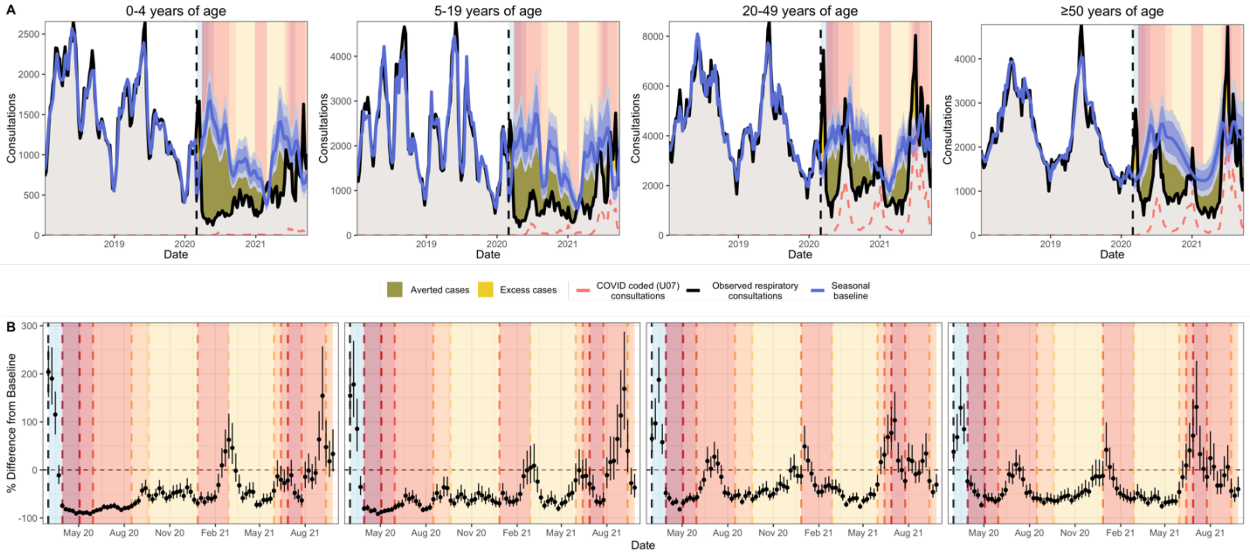

Figure S10. All-cause respiratory general practitioner encounters in all age groups.
A. Weekly all-cause respiratory encounters by age group (black), relative to all-cause respiratory encounters expected in the absence of COVID-19 (blue band). The blue band is the 95% prediction interval of the projected seasonal baseline. The red dashed line is the number of COVID-coded encounters. The black vertical dashed line indicates the start of the model prediction period (1 March 2020), and panel colors indicate the pre-lockdown period (light blue: 1 – 26 March 2020) and lockdown alert levels from March 2020 to September 2021 (Table 1). Panels are shaded according to the stringency of lockdown measures: red to orange to yellow. The area between the projected seasonal baseline and observed consultations is shaded green when observed consultations are below baseline (“averted cases”) and shaded yellow when observed consultations are above baseline (“excess cases”). **B.** Weekly observed percent difference from seasonal baseline (95% confidence interval) by age group.

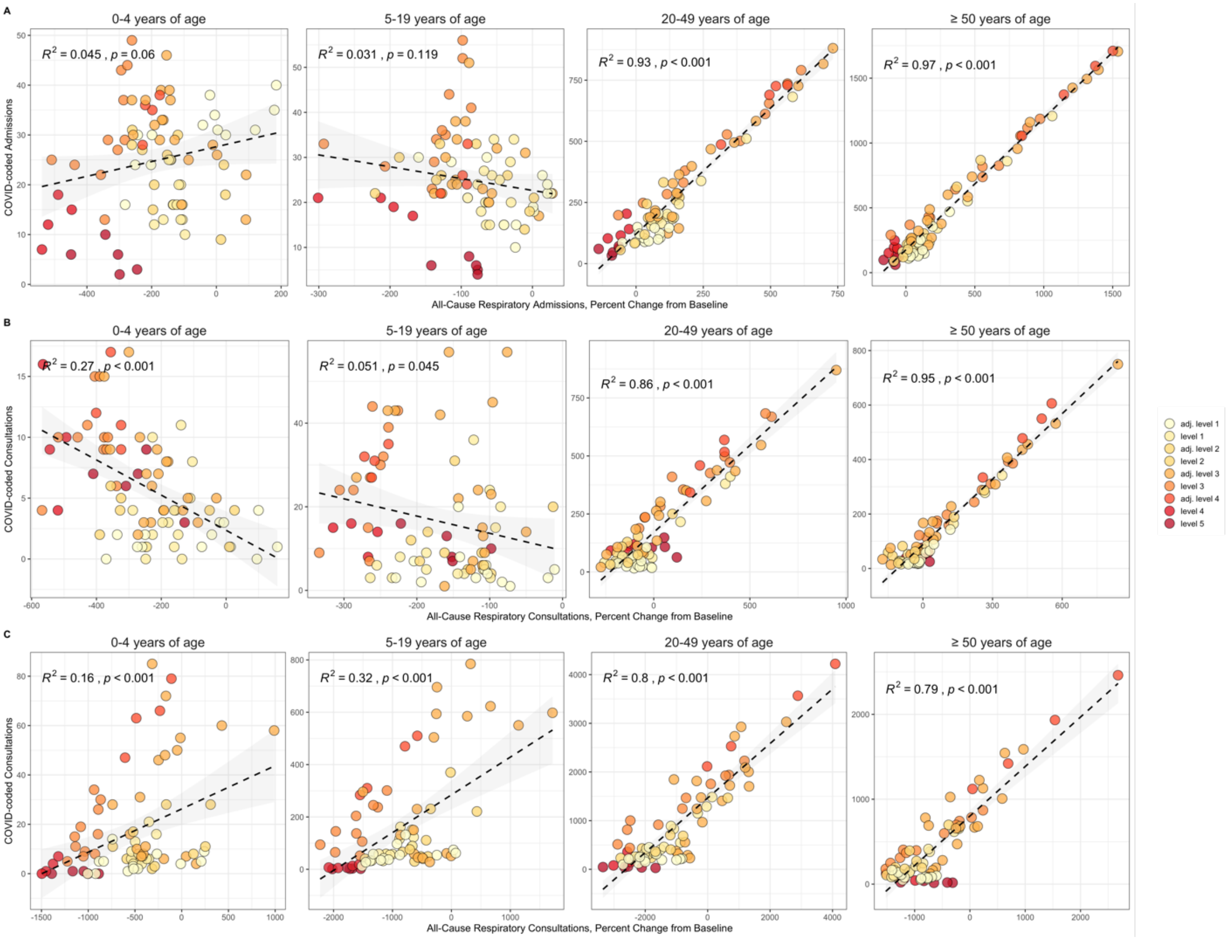

Figure S11. In adult age groups, weekly COVID-19-coded encounters strongly correlate with the percent change from baseline in all-cause respiratory encounters.

Correlations between weekly COVID-19 coded encounters and the change in baseline for all-cause respiratory encounters by age group and clinical severity: **A.** inpatient, **B.** outpatient – emergency department, and **C.** outpatient – general practitioner. Point colors indicate lockdown alert levels from March 2020 to September 2021 (Table 1) and are shaded according to the stringency of lockdown measures: red to orange to yellow.

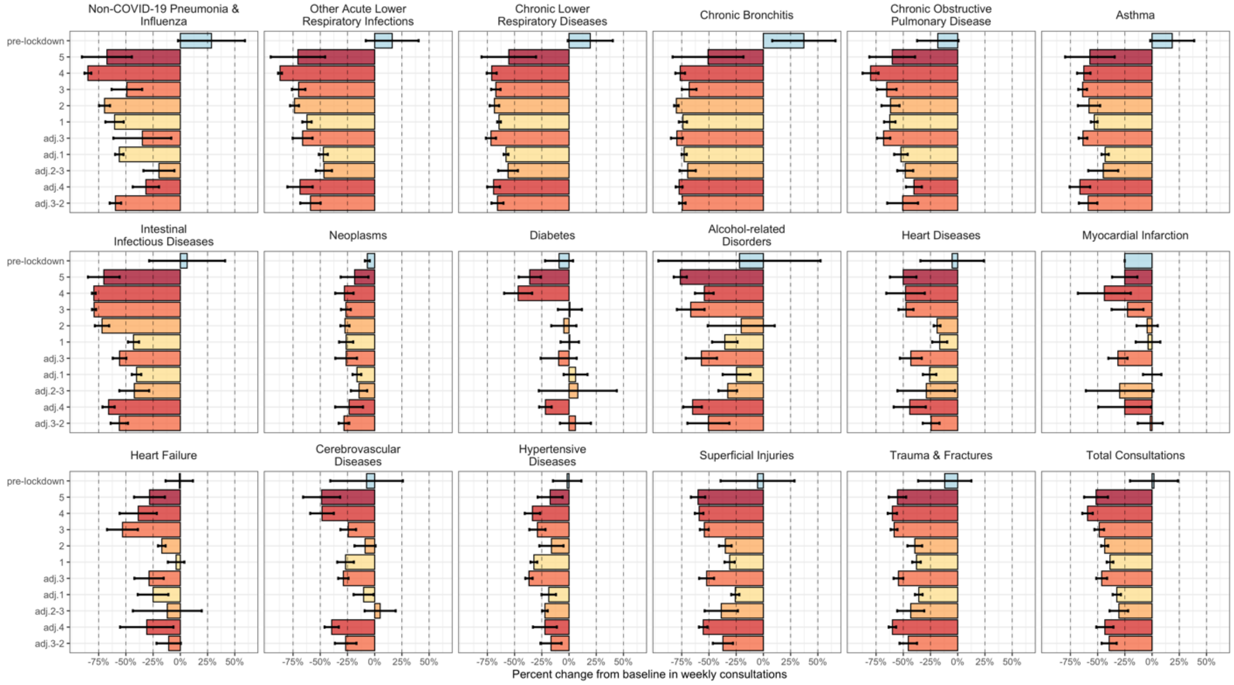

Figure S12. Percent change in weekly hospitalizations for total admissions and non-COVID-19 diagnosis groups, relative to baseline numbers expected in the absence of COVID-19.
The average percent change from baseline in weekly consultations during ten phases of the COVID-19 pandemic in South Africa: pre-lockdown (1 – 26 March 2020) and lockdown alert levels from March 2020 to September 2021 (Table 1). Bars are shaded according to the stringency of lockdown measures: red to orange to yellow. Vertical dashed lines indicate increases or reductions relative to the projected baseline number of encounters.

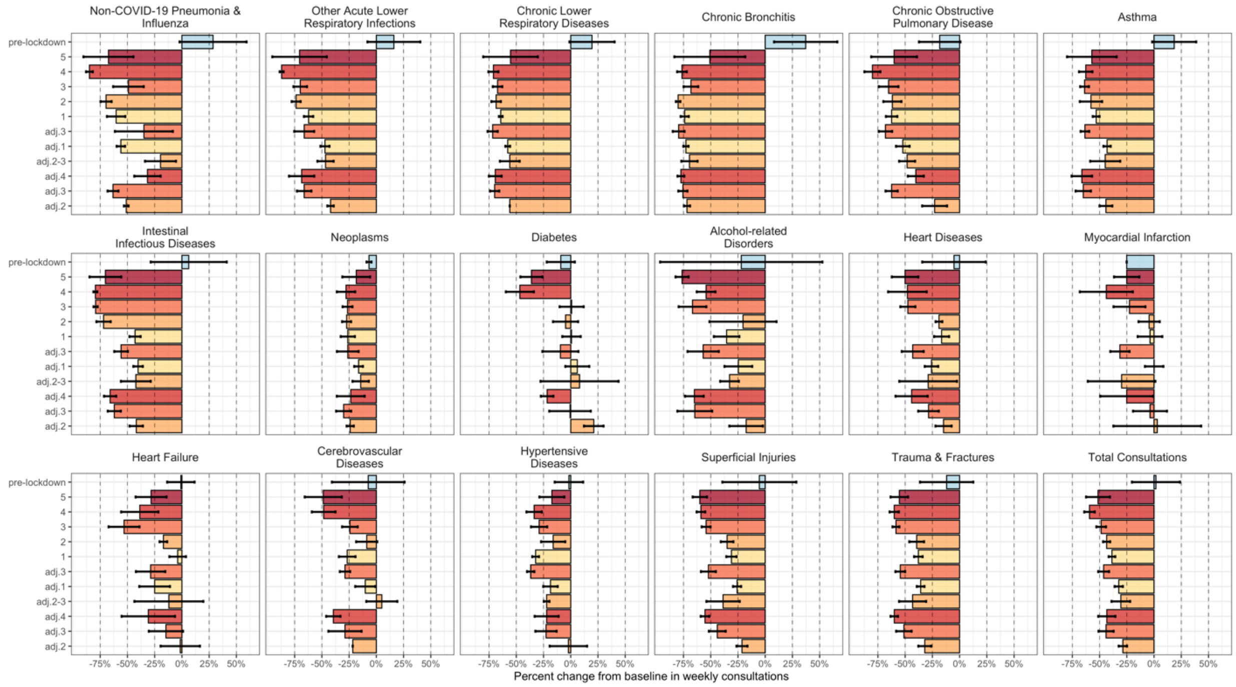

Figure S13. Percent change in weekly emergency department consultations for total consultations and non-COVID-19 diagnosis groups, relative to baseline numbers expected in the absence of COVID-19.
The average percent change from baseline in weekly consultations during ten phases of the COVID-19 pandemic in South Africa: pre-lockdown (1 – 26 March 2020) and lockdown alert levels from March 2020 to September 2021 (Table 1). Bars are shaded according to the stringency of lockdown measures: red to orange to yellow. Vertical dashed lines indicate increases or reductions relative to the projected baseline number of encounters.

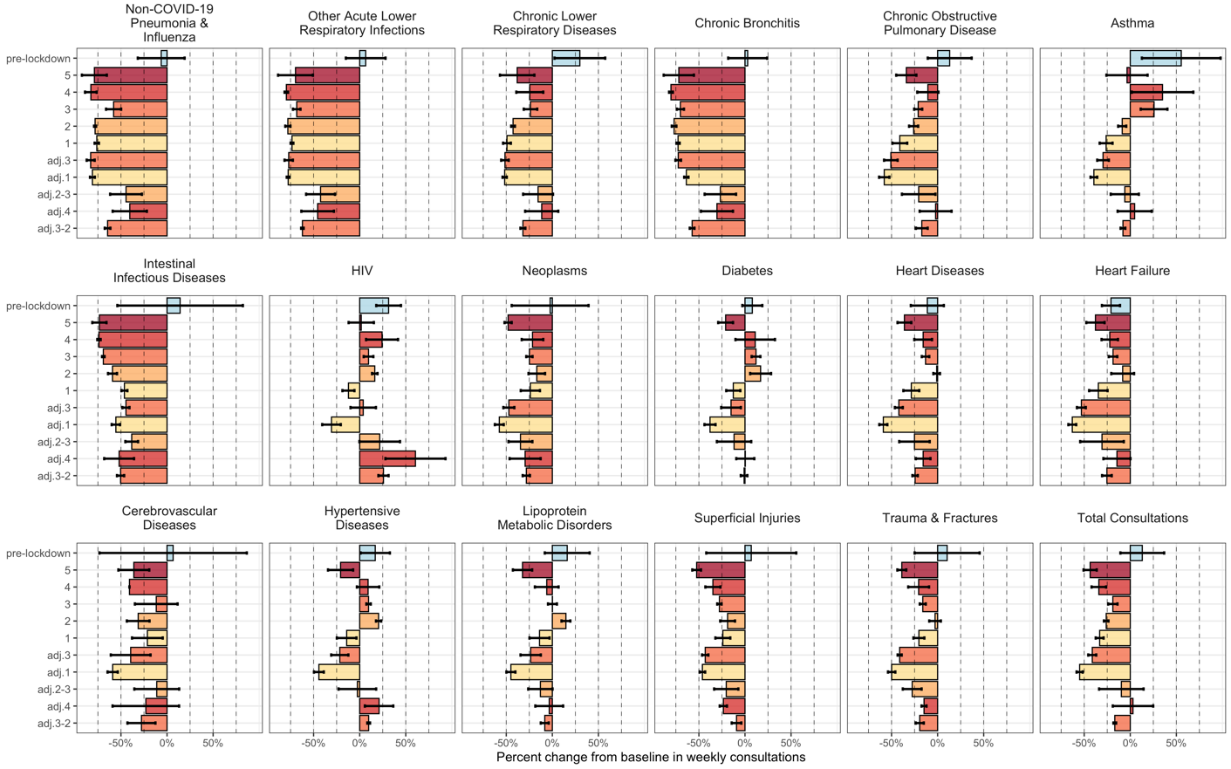

Figure S14. Percent change in weekly general practitioner consultations for total consultations and non-COVID-19 diagnosis groups, relative to baseline numbers expected in the absence of COVID-19.
The average percent change from baseline in weekly consultations during ten phases of the COVID-19 pandemic in South Africa: pre-lockdown (1 – 26 March 2020) and lockdown alert levels from March 2020 to September 2021 (Table 1). Bars are shaded according to the stringency of lockdown measures: red to orange to yellow. Vertical dashed lines indicate increases or reductions relative to the projected baseline number of encounters.

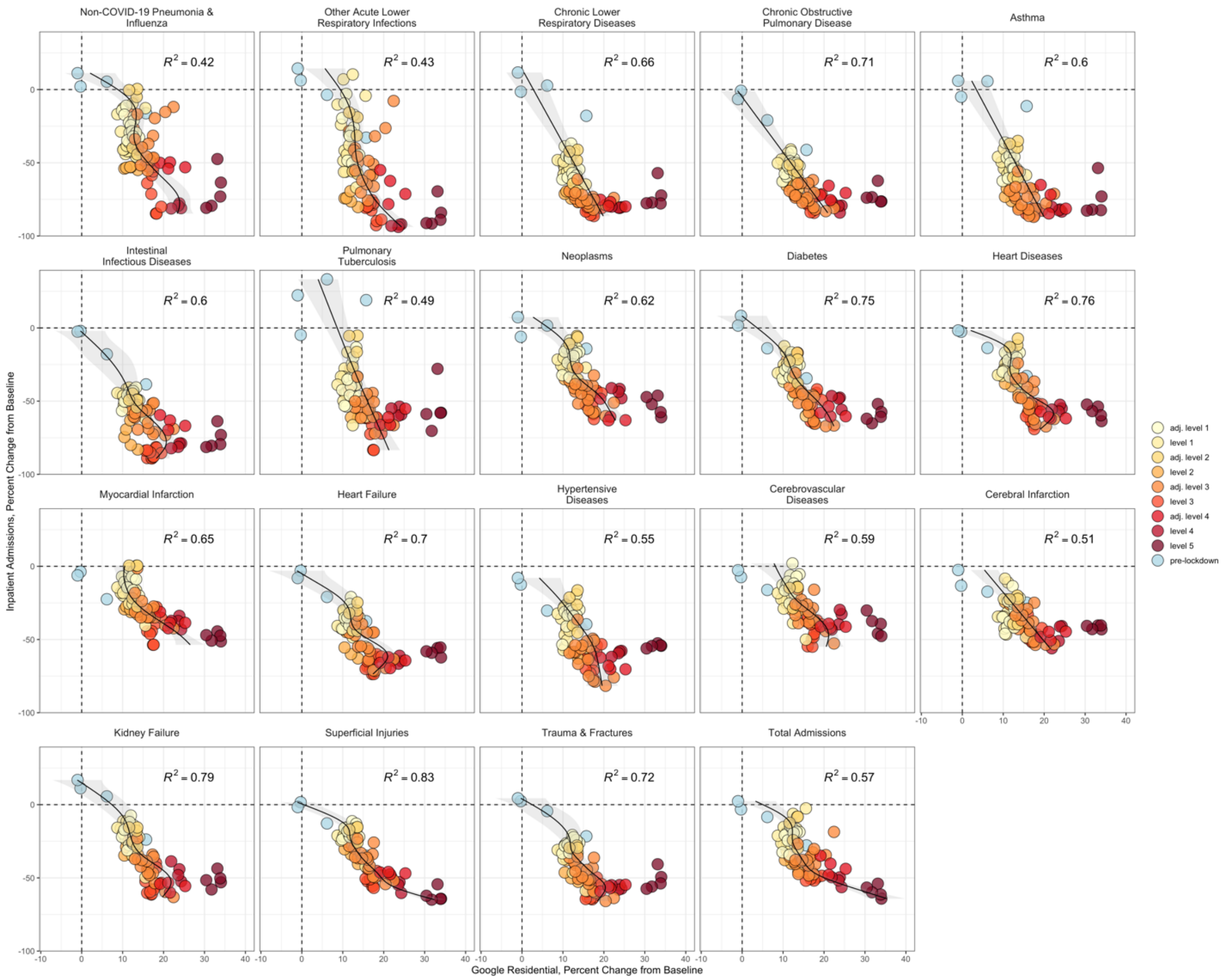

Figure S15. Visits to residential locations are associated with reduced admissions for non-COVID-19 conditions. Relationships between the weekly percent change from baseline in the Google Residential metric and the weekly percent change from baseline in total admissions and non-COVID-19 admissions. Point colors indicate the pre-lockdown period (light blue: 1- 26 March 2020) and lockdown alert levels from March 2020 to September 2021 (Table 1). Points are shaded according to the stringency of lockdown measures: red to orange to yellow. Generalized additive models (GAMs) were used to identify non-linear relationships between the Google Residential metric and inpatient admissions for each diagnosis group. GAM adjusted R^2 values are in the top right of each facet.

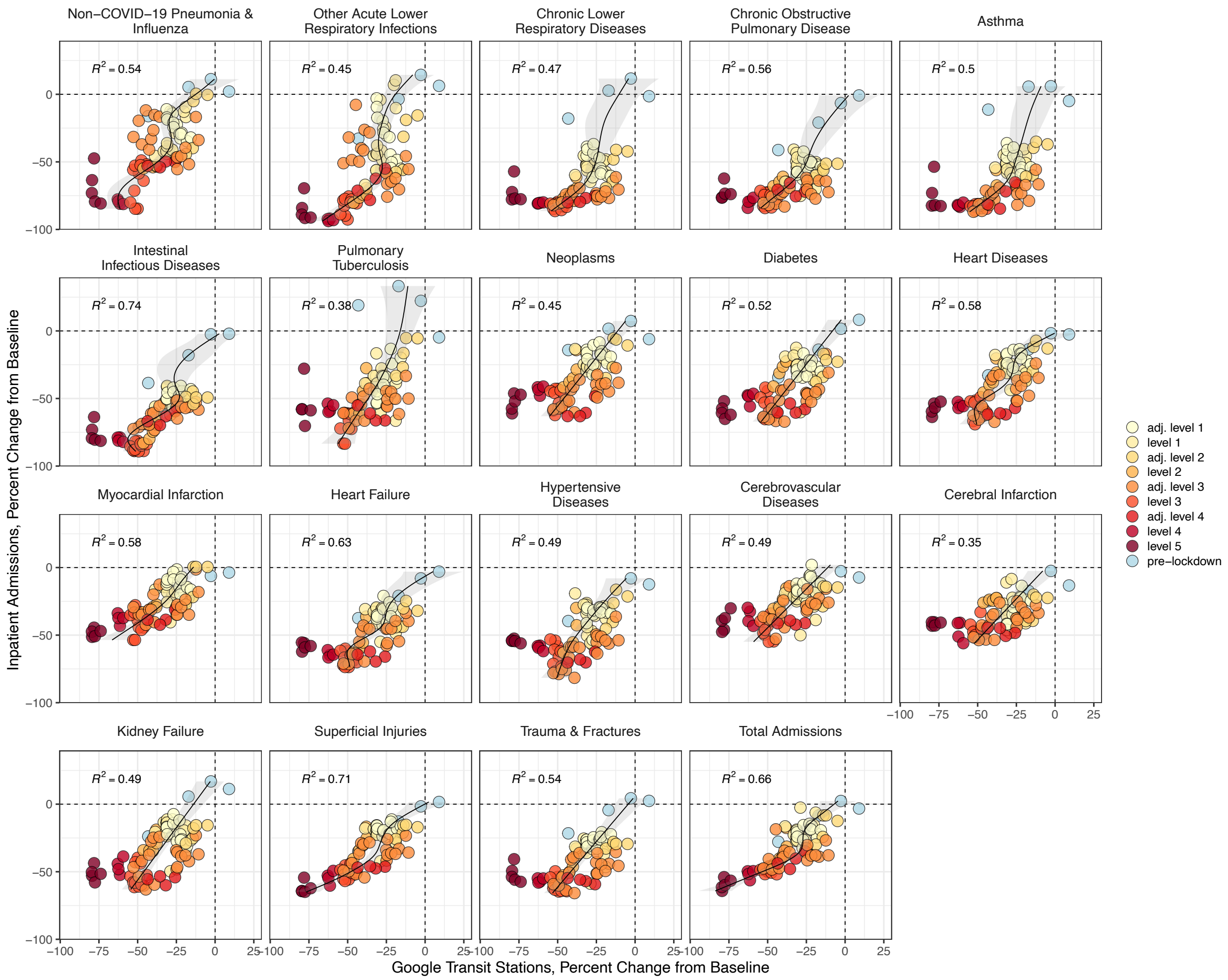

Figure S16. Visits to transit stations are associated with increased admissions for non-COVID-19 diagnoses. Relationships between the weekly percent change from baseline in the Google Transit Stations metric and the weekly percent change from baseline in non-COVID-19 admissions. Point colors indicate the pre-lockdown period (light blue: 1 – 26 March 2020) and lockdown alert levels from March 2020 to September 2021 (Table 1). Points are shaded according to the stringency of lockdown measures: red to orange to yellow. Generalized additive models (GAMs) were used to identify non-linear relationships between the Google Transit Stations metric and inpatient admissions for each diagnosis group. GAM adjusted R^2 values are in the top left of each facet.

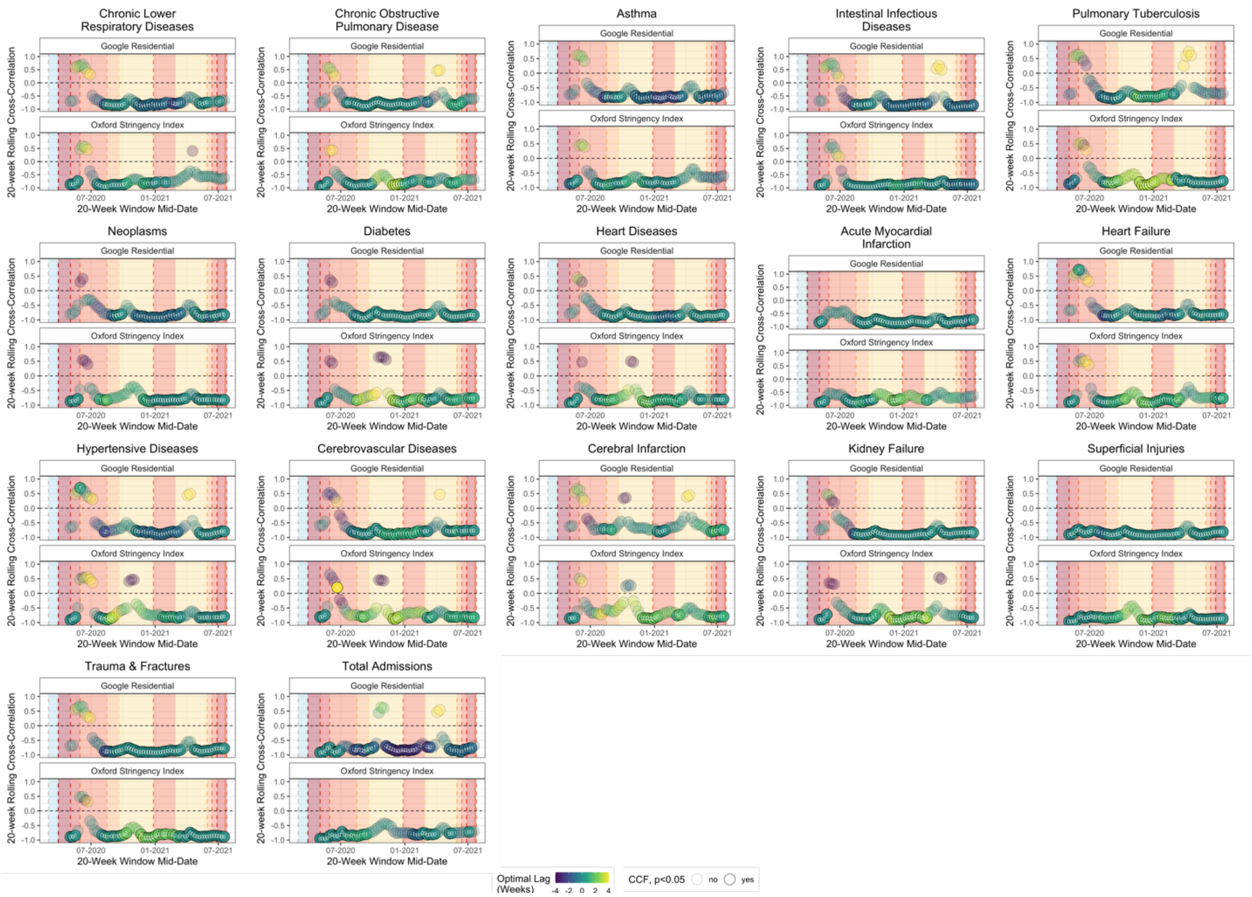

Figure S17. Rolling cross-correlations between non-COVID-19 admissions and visits to residential locations or COVID-19 policy stringency during the COVID-19 pandemic.

Points represent the maximum coefficient values for 20-week rolling cross-correlations between admissions and the Google Residential (GR) metric or the Oxford Policy Stringency Index (OPSI). Point color and the number within each point indicate the lag in weeks corresponding to the maximum cross-correlation coefficient value for each 20-week time period ("optimal lag"). Negative values indicate that GR or OPSI lead admissions, and positive values indicate that GR or OPSI lag behind admissions. A lag of 0 indicates that the time series are in phase. Point transparency indicates statistical significance of the cross-correlation coefficient (yes: solid, no: transparent). Panel colors indicate the pre-lockdown period (light blue: 1 – 26 March 2020) and lockdown alert levels from March 2020 to September 2021 (Table 1). Panels are shaded according to the stringency of lockdown measures: red to orange to yellow.

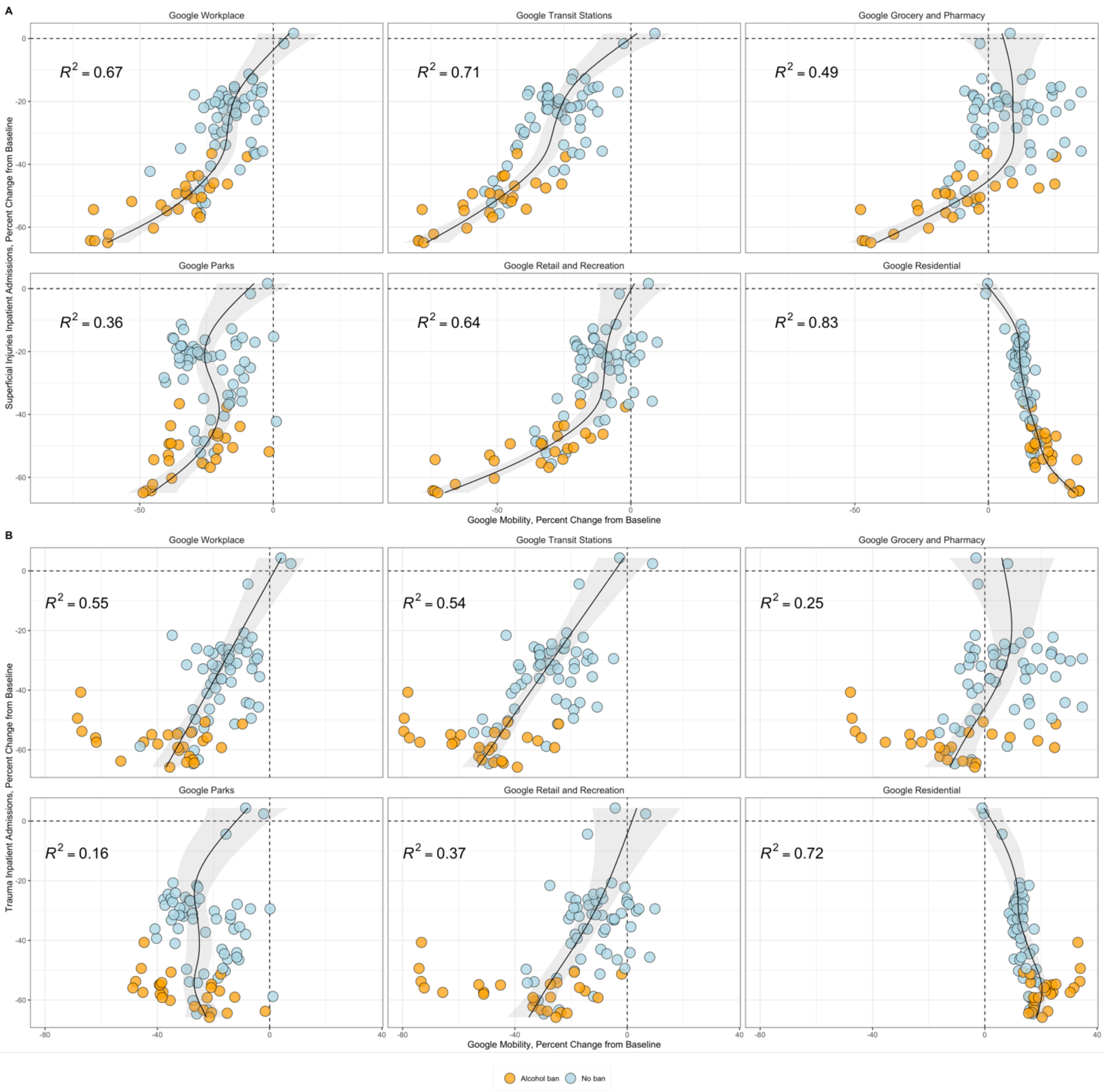

Figure S18. Associations between Google mobility metrics and the weekly percent change from baseline for diagnoses coded as A. superficial injuries and B. trauma or fractures.

Point colors indicate weeks during which alcohol sales were banned (first ban: 27 March – 1 June 2020, second ban: 12 July – 17 August 2020, third ban: 28 December 2020 – 1 February 2021, fourth ban: 28 June – 25 July 2021). Generalized additive models (GAMs) were used to identify non-linear relationships between Google Mobility metrics and inpatient admissions. GAM adjusted R^2 values are in the top left of each facet.

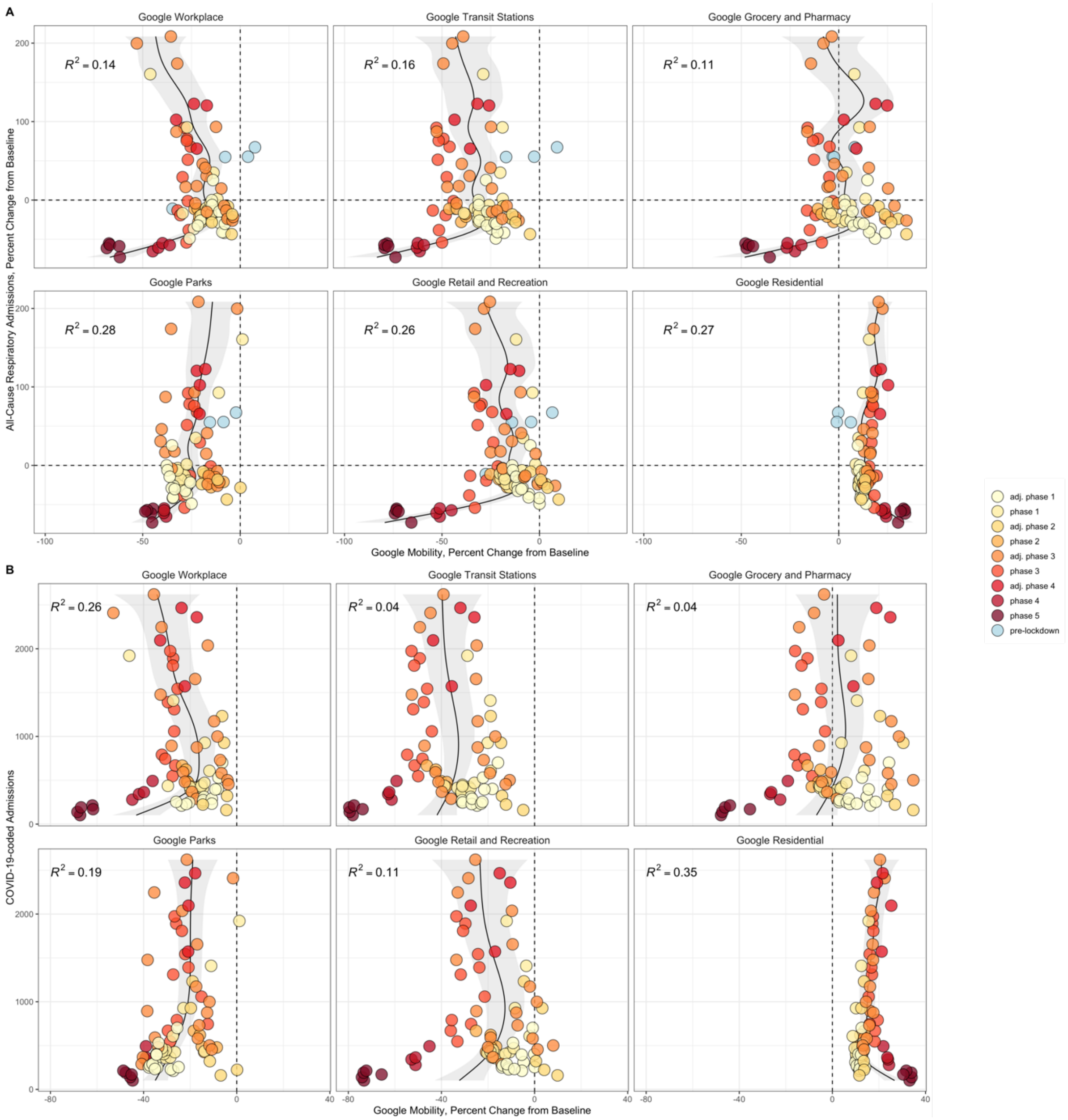

Figure S19. Non-linear associations between Google mobility metrics and respiratory admissions in individuals aged ≥ 5 years.

A. Relationships between the weekly percent change from baseline for Google mobility metrics and the weekly percent change from baseline for all-cause respiratory admissions (including COVID-19) among individuals aged ≥ 5 years. **B.** Relationships between the weekly percent change from baseline for Google mobility metrics and the weekly number of COVID-19-coded admissions among individuals aged ≥ 5 years. Point colors indicate the pre-lockdown period (light blue: 1 – 26 March 2020) and lockdown alert levels from March 2020 to September 2021 (Table 1). Points are shaded according to the stringency of lockdown measures: red to orange to yellow. Generalized additive models (GAMs) were used to identify non-linear relationships between Google Mobility metrics and inpatient admissions. GAM adjusted R^2 values are in the top left of each facet.

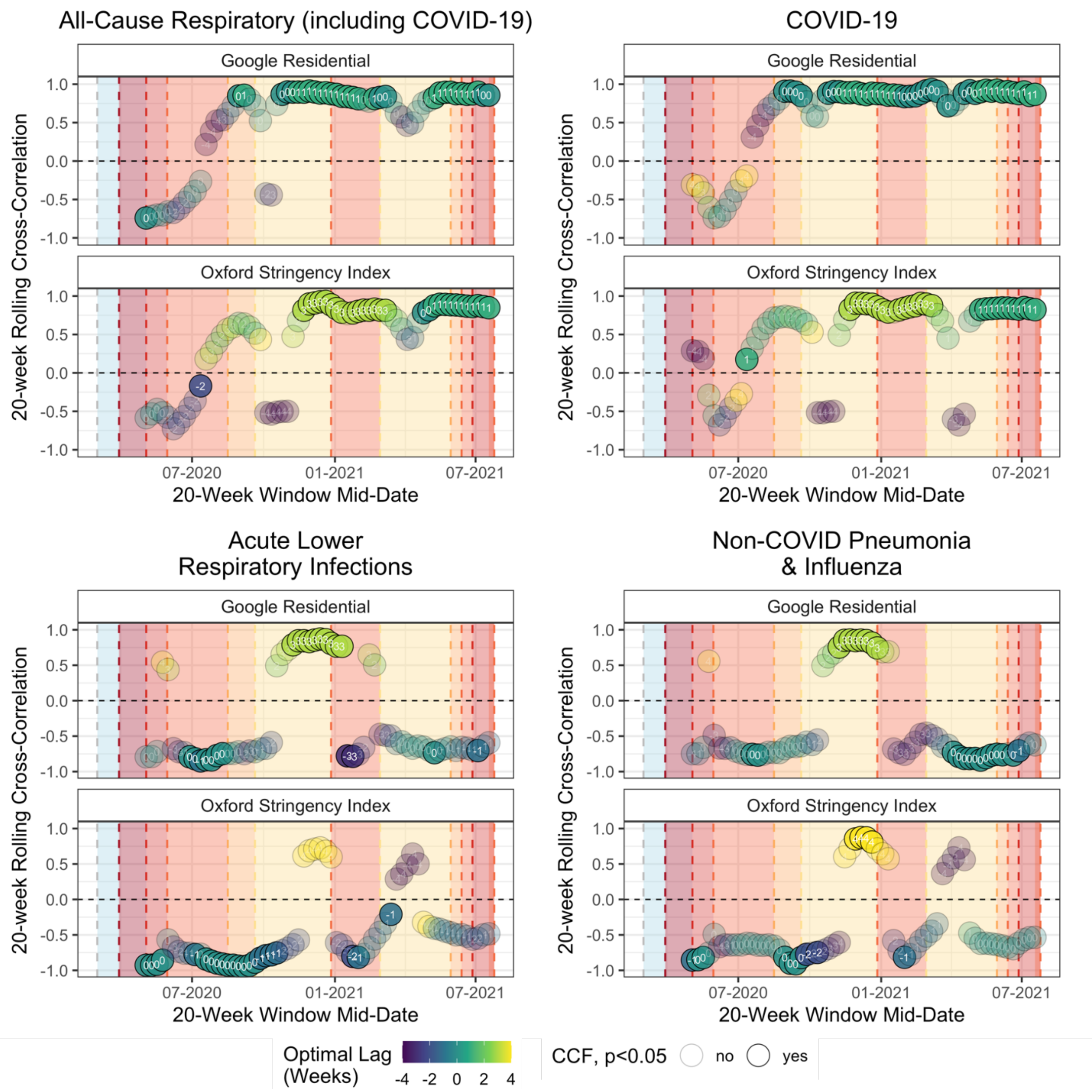

Figure S20. Rolling cross-correlations between respiratory admissions and visits to residential locations or policy stringency during the COVID-19 pandemic.

Points represent the maximum coefficient values for 20-week rolling cross-correlations between respiratory admissions (all-cause respiratory, including COVID-19; COVID-19 only; acute lower respiratory infections; non-COVID-19 pneumonia and influenza) and the Google Residential (GR) metric or the Oxford Policy Stringency Index (OPSI). Point color and the number within each point indicate the lag in weeks corresponding to the maximum cross-correlation coefficient value for each 20-week time period ("optimal lag"). Negative values indicate that GR or OPSI lead admissions, and positive values indicate that GR or OPSI lag behind admissions. A lag of 0 indicates that the time series are in phase. Point transparency indicates statistical significance of the cross-correlation coefficient (yes: solid, no: transparent). Panel colors indicate the pre-lockdown period (light blue: 1 – 26 March 2020) and lockdown alert levels from March 2020 to September 2021 (Table 1). Panels are shaded according to the stringency of lockdown measures: red to orange to yellow.

Table S1. Diagnoses and associated ICD-10 codes[a] analyzed for each type of consultation: inpatient, outpatient - emergency department, and outpatient - general practitioner.

Diagnosis	ICD-10 codes	Inpatient	Outpatient Emergency Department	Outpatient General Practitioner
**Respiratory conditions**				
All-cause respiratory illness, including COVID-19	J00-J99, U07.1, U07.2	X	X	X
Pneumonia and influenza	J09-J18	X	X	X
Other acute lower respiratory infections	J20-J22	X	X	X
Chronic lower respiratory diseases	J40-J47	X	X	X
Chronic bronchitis	J40-42		X	X
Chronic obstructive pulmonary disease	J44	X	X	X
Asthma	J45	X	X	X
**Non-respiratory conditions**				
Intestinal infectious diseases	A00-A09	X	X	X
Pulmonary tuberculosis	A15-A16	X		
HIV	B20-B24			X
Neoplasms	C00-D49	X	X	X
Diabetes mellitus	E10-E14	X	X	X
Alcohol-related disorders	F10		X	
Heart diseases	I00-I09, I11, I13, I20-I51	X	X	X
Acute myocardial infarction	I21	X	X	
Heart failure	I50	X	X	X
Hypertensive diseases	I10-I16	X	X	X
Cerebrovascular diseases	I60-I69	X	X	X
Cerebral infarction	I63	X		
Disorders of lipoprotein metabolism	E78			X
Acute kidney failure and chronic kidney disease	N17-N19	X		
Superficial injuries	S00-S99	X	X	X
Trauma and fractures	T07-T88	X	X	X

[a]Diagnosis codes for Alzheimer's disease (G30), tuberculosis of other organs (A17-A19), and traffic and non-traffic accidents (V) were considered but encounters were too few to perform downstream analyses.

Table S2. Coding for individual policy indicators in the Oxford COVID-19 Government Response Tracker (OxCGRT) database[4]**.** The stringency index combines indicators C1 to C8 and H1. Each indicator includes 0 values for dates when no policy measures were taken.

**Containment and closure policies**		
**C1**	School closing	1 - recommend closing or all schools open with alterations<br>2 - require closing for only some levels or categories<br>3 - require closing for all levels
**C2**	Workplace closing	1 - recommend closing or work from home<br>2 - require closing or work from home for some sectors<br>3 - require closing or work from home for all-but-essential workplaces
**C3**	Cancel public events	1 - recommend cancelling<br>2 - require cancelling
**C4**	Restrictions on gatherings	1 - > 1000 people<br>2 - 101-1000 people<br>3 - 11-100 people<br>4 - < 11 people
**C5**	Close public transport	1 - recommend closing or significantly reduce volume/route/means of transport available<br>2 - require closing or prohibit most citizens from using it
**C6**	Stay at home requirements	1 - recommend not leaving house<br>2 - require not leaving house with exceptions for daily exercise, grocery shopping, and 'essential' trips<br>3 - require not leaving house with minimal exceptions
**C7**	Restrictions on internal movement	1 - recommend not to travel between regions/cities<br>2 - internal movement restrictions in place
**C8**	International travel controls	1 - screening arrivals<br>2 - quarantine arrivals from some or all regions<br>3 - ban arrivals from some regions<br>4 - ban on all regions or total border closure
**Health system policies**		
**H1**	Public information campaigns	1 - public officials urging caution about COVID-19<br>2 - coordinated public information campaign across traditional and social media

Table S3. Percent change in emergency department consultations relative to baseline numbers expected in the absence of COVID-19. The average percent change from baseline in weekly consultations at a private healthcare group during ten phases of the COVID-19 pandemic in South Africa: pre-lockdown (1 - 26 March 2020) and lockdown alert levels from March 2020 to September 2021 (Table 1). Brackets include 95% confidence intervals for mean estimates.

**Diagnosis**	**Pre-lockdown**	**Level 5**	**Level 4**	**Level 3**	**Level 2**	**Level 1**	**Adjusted Level 3**	**Adjusted Level 1**	**Adjusted Levels 2-3**	**Adjusted Level 4**	**Adjusted Levels 3-2**
**Respiratory conditions**											
All-cause respiratory	37.6 [-15.8, 91]	-53.3 [-73.3, -33.2]	-67.3 [-79.1, -55.6]	-16.7 [-37.6, 4.2]	-68.6 [-72.4, -64.7]	-57.2 [-70.9, -43.5]	-29.4 [-75.1, 16.3]	-65.6 [-71.6, -59.5]	-9 [-50.1, 32.1]	9.8 [-24.6, 44.1]	-52.8 [-64.6, -41]
Pneumonia and influenza	28.6 [-2.1, 59.3]	-67.4 [-90.4, -44.5]	-84.9 [-88, -81.8]	-49.1 [-63.1, -35]	-69.6 [-74.7, -64.6]	-60.4 [-68.8, -52]	-34.9 [-61.5, -8.3]	-56.1 [-59.9, -52.2]	-19.7 [-33.9, -5.4]	-31.6 [-43.7, -19.6]	-59.6 [-64.9, -54.4]
Other acute lower respiratory infections	16.1 [-8.2, 40.3]	-70.5 [-95.5, -45.5]	-86.9 [-88.7, -85.2]	-69.9 [-76.1, -63.6]	-73.6 [-77.7, -69.5]	-62.2 [-66.5, -57.9]	-66.2 [-75.3, -57.1]	-47.2 [-51.3, -43]	-46.6 [-54.1, -39.2]	-68.6 [-80.2, -57]	-59 [-68.4, -49.6]
Chronic lower respiratory diseases	19.5 [-1.2, 40.2]	-55.3 [-80.4, -30.3]	-71 [-75.6, -66.4]	-67.2 [-71.6, -62.9]	-68.5 [-72.9, -64.2]	-64.3 [-66, -62.6]	-71.7 [-76.4, -67]	-57.9 [-60, -55.7]	-56 [-65.1, -46.9]	-69.3 [-75.2, -63.5]	-65.7 [-71.2, -60.2]
Chronic bronchitis	37.2 [8.1, 66.2]	-50.8 [-83.4, -18.3]	-76.4 [-80.8, -72]	-68.2 [-74.9, -61.4]	-79.9 [-82, -77.9]	-73.9 [-77.6, -70.1]	-79.5 [-84.9, -74.1]	-72.8 [-75.1, -70.4]	-69.5 [-77, -62.1]	-77.4 [-80.5, -74.3]	-74.4 [-77.4, -71.5]
Chronic obstructive pulmonary disease	-18.3 [-37.3, 0.7]	-60.1 [-81.1, -39.1]	-80.1 [-87.4, -72.7]	-65.2 [-74.3, -56.2]	-61.7 [-70, -53.5]	-62.4 [-67.7, -57.2]	-68.1 [-74.2, -62]	-52.2 [-58.4, -46]	-48.2 [-55.5, -40.9]	-40.2 [-47.5, -32.9]	-50.6 [-64.7, -36.4]
Asthma	18.5 [-1.7, 38.7]	-57.1 [-79.9, -34.3]	-62.7 [-68.9, -56.6]	-63.8 [-67.8, -59.7]	-57.9 [-68.2, -47.7]	-53.1 [-56.3, -50]	-63.4 [-67.4, -59.5]	-43.1 [-46.4, -39.8]	-44.8 [-58.6, -31]	-66.3 [-75.7, -56.9]	-58.7 [-67.2, -50.2]
**Non-respiratory conditions**											
Intestinal infectious Diseases	6.3 [-28.6, 41.2]	-70.3 [-84.8, -55.7]	-79.5 [-81.2, -77.7]	-79.2 [-81.1, -77.4]	-71.9 [-78.5, -65.4]	-43 [-48.2, -37.9]	-55.9 [-62.1, -49.6]	-40.2 [-44.6, -35.9]	-42.3 [-56, -28.6]	-65.9 [-71.5, -60.3]	-56 [-64, -48.1]
Neoplasms	-6.7 [-9, -4.5]	-18.5 [-31.2, -5.7]	-27.8 [-36.3, -19.4]	-26.4 [-31, -21.9]	-27.3 [-31.4, -23.2]	-26.2 [-32.7, -19.7]	-26.2 [-36.2, -16.3]	-16.3 [-20.3, -12.3]	-14.4 [-21.9, -6.9]	-23.5 [-36.3, -10.7]	-28.2 [-33.1, -23.4]
Diabetes	-9.2 [-22, 3.7]	-36.1 [-46.2, -26]	-46.7 [-59.6, -33.9]	0.8 [-10.2, 11.9]	-4.8 [-16.3, 6.8]	0.7 [-7.8, 9.1]	-9.5 [-26, 7]	6 [-5, 17]	8.1 [-27.8, 43.9]	-21.7 [-27.4, -15.9]	5.9 [-8.4, 20.1]
Alcohol-related disorders	-22 [-96.5, 52.5]	-76.3 [-82.3, -70.3]	-54.3 [-62.8, -45.7]	-66.7 [-79.4, -54]	-20.3 [-51.1, 10.4]	-35.4 [-47.1, -23.7]	-56.9 [-71.3, -42.6]	-24.7 [-37.4, -11.9]	-32.7 [-41.3, -24.2]	-65 [-73.6, -56.5]	-50.5 [-69.8, -31.2]
Heart diseases	-5.1 [-34.3, 24]	-50.1 [-62.3, -37.9]	-47.9 [-65.6, -30.2]	-47.5 [-54.4, -40.6]	-19 [-22, -15.9]	-16.6 [-23.5, -9.6]	-43.1 [-53.1, -33]	-25.8 [-32, -19.7]	-28.9 [-55.3, -2.4]	-44.1 [-58.8, -29.3]	-24.4 [-32.1, -16.7]
Acute myocardial infarction	-25.1 [-25.1, -25.1]	-25.1 [-36.7, -13.5]	-43.8 [-68.2, -19.5]	-22.6 [-37.2, -7.9]	-4.5 [-14.2, 5.2]	-3.7 [-15, 7.6]	-31.3 [-40.2, -22.5]	0.1 [-8.3, 8.6]	-29.8 [-60.8, 1.2]	-25.1 [-49.4, -0.8]	-1.7 [-13.2, 9.8]
Heart failure	-1 [-13.6, 11.6]	-28.3 [-42.5, -14.1]	-38.7 [-55.8, -21.6]	-53.1 [-67.3, -38.9]	-17 [-20.5, -13.5]	-3.9 [-11.5, 3.7]	-28.7 [-42.2, -15.3]	-25 [-39.1, -10.9]	-12 [-43.6, 19.7]	-30.8 [-55.3, -6.3]	-10.7 [-21.9, 0.5]
Cerebrovascular diseases	-7.4 [-40.8, 25.9]	-48.7 [-65.7, -31.7]	-48.4 [-59.2, -37.5]	-24.3 [-31.4, -17.1]	-8.9 [-18.5, 0.8]	-26.8 [-34.4, -19.1]	-28.8 [-33.5, -24.1]	-10.2 [-19.4, -1]	5 [-9.2, 19.3]	-39.5 [-46, -32.9]	-26.7 [-36.6, -16.7]
Hypertensive	-1.7 [-14.8, 11.4]	-17.4 [-28.8, -5.9]	-33.6 [-40.7, -26.6]	-29 [-36.5, -21.6]	-16.2 [-27.2, -5.1]	-32.3 [-35.4, -29.2]	-36.8 [-40.1, -33.4]	-18.6 [-25.3, -12]	-22.1 [-24.7, -19.6]	-22.1 [-33.2, -11.1]	-16.6 [-26.2, -6.9]

diseases											
Superficial injuries	-5.4 [-39.3, 28.6]	-60 [-66.6, -53.4]	-59 [-62.9, -55.1]	-54.3 [-58.3, -50.2]	-35 [-40.8, -29.2]	-31 [-35.7, -26.3]	-52.1 [-59, -45.3]	-25.8 [-29.5, -22.1]	-38.7 [-54, -23.4]	-55.3 [-59.2, -51.4]	-37.2 [-46.4, -27.9]
Trauma and fractures	-11.9 [-36.4, 12.6]	-55.3 [-63.4, -47.3]	-60 [-64.3, -55.8]	-58.4 [-61.6, -55.3]	-39.4 [-46.2, -32.7]	-37.9 [-41.8, -34]	-54.5 [-58.9, -50]	-35.8 [-39.3, -32.3]	-43.2 [-55.6, -30.8]	-60 [-63.4, -56.6]	-45.4 [-53.5, -37.4]
**Total consultations**	1.7 [-20.3, 23.8]	-51.4 [-62.4, -40.5]	-59.3 [-64.1, -54.5]	-48.5 [-53, -44.1]	-43.5 [-46.8, -40.3]	-38.6 [-41.7, -35.6]	-46.3 [-51.3, -41.3]	-32.4 [-36.2, -28.6]	-30.5 [-39, -21.9]	-43.4 [-51.2, -35.5]	-39.4 [-46.3, -32.5]

Table S4. Percent change in general practitioner consultations relative to baseline numbers expected in the absence of COVID-19. The average percent change from baseline in weekly consultations at a private healthcare group during ten phases of the COVID-19 pandemic in South Africa: pre-lockdown (1 - 26 March 2020) and lockdown alert levels from March 2020 to September 2021 (Table 1). Brackets include 95% confidence intervals for mean estimates.

Diagnosis	Pre-lockdown	Level 5	Level 4	Level 3	Level 2	Level 1	Adjusted Level 3	Adjusted Level 1	Adjusted Levels 2-3	Adjusted Level 4	Adjusted Levels 3-2
**Respiratory conditions**											
All-cause respiratory	37.5 [-9.2, 84.2]	-66.8 [-78.5, -55.2]	-64.9 [-66.9, -62.8]	-27.7 [-43.4, -12]	-59.9 [-65.7, -54.1]	-52.6 [-56.6, -48.7]	-58.1 [-72.8, -43.5]	-67.8 [-71.5, -64]	9.3 [-41.6, 60.1]	28.2 [-35.6, 92.1]	-5.8 [-28, 16.4]
Pneumonia and influenza	-6.3 [-31.7, 19.1]	-78.9 [-92.4, -65.3]	-82.6 [-89, -76.3]	-58 [-66.2, -49.8]	-78.1 [-79.2, -76.9]	-76.2 [-78.6, -73.9]	-82.9 [-87.2, -78.5]	-81 [-83.6, -78.3]	-44.5 [-61.7, -27.3]	-40.4 [-59, -21.9]	-64.5 [-67.6, -61.4]
Other acute lower respiratory infections	6.5 [-15, 28]	-69.6 [-88.6, -50.7]	-79.6 [-81.5, -77.7]	-68.3 [-72.4, -64.2]	-77.9 [-80.7, -75.1]	-73.4 [-74.9, -71.9]	-77 [-81.5, -72.5]	-77.6 [-79.1, -76.1]	-42.5 [-58.1, -26.8]	-45.6 [-63.3, -27.9]	-62.1 [-63.5, -60.8]
Chronic lower respiratory diseases	29.9 [2.3, 57.4]	-38.2 [-56.8, -19.5]	-24.5 [-39.1, -9.9]	-23.7 [-31, -16.4]	-42.6 [-44.7, -40.6]	-49.2 [-53.4, -45]	-51.4 [-55.5, -47.3]	-51.7 [-54, -49.4]	-15.5 [-31.7, 0.7]	-11.5 [-29.5, 6.5]	-31.9 [-34.6, -29.3]
Chronic bronchitis	2.9 [-18.2, 24.1]	-71.8 [-88.2, -55.4]	-80.3 [-82.4, -78.3]	-70.1 [-74, -66.2]	-76.9 [-79.4, -74.4]	-72.6 [-74.4, -70.9]	-72.4 [-75.4, -69.4]	-63.6 [-65.9, -61.3]	-26.7 [-43.8, -9.7]	-30.4 [-47.8, -13]	-57.3 [-59.9, -54.8]
Chronic obstructive pulmonary disease	13.1 [-10.7, 36.9]	-34 [-44.9, -23]	-10.6 [-22.1, 0.9]	-21.1 [-25.5, -16.7]	-26 [-31, -21.1]	-41.1 [-49.1, -33]	-50.8 [-58.1, -43.4]	-57.9 [-63.5, -52.3]	-20.5 [-38.7, -2.3]	-2.2 [-19.5, 15.1]	-17.2 [-23.6, -10.8]
Asthma	55.3 [12.6, 98]	-3.5 [-25.9, 18.8]	35 [1.6, 68.4]	25.8 [11.3, 40.3]	-8.8 [-13.1, -4.4]	-26.2 [-33.2, -19.2]	-29.6 [-35.9, -23.2]	-39.5 [-43.1, -35.9]	-5.9 [-21.2, 9.3]	4.7 [-13.7, 23.2]	-8 [-10.5, -5.4]
**Non-respiratory conditions**											
Intestinal infectious diseases	14.2 [-53.9, 82.3]	-73.4 [-81.1, -65.7]	-73.8 [-75.7, -72]	-69.1 [-70.5, -67.7]	-59.2 [-64, -54.3]	-46.2 [-49.5, -43]	-44.6 [-48.4, -40.7]	-55.5 [-60, -51]	-38.3 [-45.2, -31.4]	-52 [-68.2, -35.8]	-50.4 [-54.3, -46.5]
HIV	31.4 [18, 44.8]	1.7 [-12, 15.4]	24.3 [6.9, 41.6]	9.6 [4.4, 14.8]	16.4 [13.4, 19.3]	-12.2 [-18.7, -5.7]	3.8 [-9.9, 17.6]	-30.6 [-40.7, -20.5]	21.7 [-0.3, 43.8]	60.5 [27.9, 93.1]	25.8 [20.3, 31.3]
Neoplasms	-2.4 [-44.1, 39.3]	-48 [-51.9, -44.1]	-21.5 [-33.2, -9.8]	-24.9 [-28.3, -21.5]	-16.9 [-25.8, -7.9]	-23.8 [-34.3, -13.4]	-47.2 [-53.1, -41.2]	-57.5 [-62.4, -52.7]	-34.6 [-47.5, -21.6]	-29.5 [-46.4, -12.7]	-28.2 [-32.2, -24.3]
Diabetes	8 [-2.9, 18.8]	-21 [-29.1, -13]	11.1 [-10.3, 32.6]	12 [7.2, 16.7]	17 [5.6, 28.3]	-12.7 [-20.4, -5]	-15.1 [-25.8, -4.5]	-37.9 [-44, -31.9]	-11.9 [-30.5, 6.8]	0.3 [-9.5, 10.1]	-1 [-4.5, 2.6]
Heart diseases	-11.2 [-29.1, 6.7]	-36 [-43.5, -28.6]	-15.7 [-25.3, -6.1]	-13.1 [-17.2, -9]	-1 [-4.6, 2.6]	-28.6 [-37.2, -19.9]	-42.1 [-46.4, -37.8]	-58.9 [-63.3, -54.5]	-25.1 [-41.7, -8.6]	-15.7 [-23.6, -7.8]	-24.4 [-27.4, -21.5]
Heart failure	-20.9 [-30.8, -11.1]	-37.8 [-47.7, -27.9]	-22.2 [-31.2, -13.3]	-18.7 [-23.3, -14.1]	-8.2 [-20.6, 4.1]	-34.6 [-44.7, -24.6]	-53.1 [-57.9, -48.2]	-62.9 [-67.2, -58.7]	-30.7 [-54.3, -7]	-14.5 [-29.1, 0.2]	-25.4 [-30.6, -20.1]
Cerebrovascular diseases	6.6 [-73.4, 86.6]	-36 [-52.8, -19.3]	-40.8 [-40.8, -40.8]	-11.7 [-34.9, 11.5]	-31.3 [-43.6, -19]	-21.3 [-38, -4.7]	-39.5 [-61, -17.9]	-59 [-64.6, -53.4]	-11.2 [-35.5, 13.2]	-23 [-59.1, 13.1]	-27.7 [-42.9, -12.6]
Hypertensive diseases	16.9 [1, 32.8]	-20.8 [-34.4, -7.1]	9.1 [-3, 21.2]	9.6 [7.2, 12]	20.5 [17.7, 23.3]	-14.1 [-24.8, -3.5]	-21.6 [-30.9, -12.3]	-44.3 [-49.6, -38.9]	-2.5 [-22.9, 17.9]	21 [5.6, 36.4]	9.7 [8.3, 11.2]
Lipoprotein metabolic disorders	16.1 [-8.2, 40.5]	-32.3 [-42.8, -21.9]	-6.1 [-18.9, 6.8]	0.1 [-4.7, 5]	14.7 [10, 19.5]	-13.9 [-24.8, -3]	-23.5 [-34.5, -12.5]	-45 [-49.8, -40.1]	-13 [-26.2, 0.2]	-3.4 [-18.4, 11.7]	-8.2 [-12.5, -3.9]

Superficial injuries	6.8 [-42, 55.6]	-52.5 [-57.2, -47.7]	-34.8 [-43, -26.7]	-27.7 [-30.1, -25.4]	-18.9 [-26.9, -10.8]	-24 [-32.1, -15.9]	-43.1 [-46.6, -39.7]	-46.2 [-49.2, -43.2]	-20.3 [-33.5, -7.2]	-23.5 [-27.4, -19.5]	-9.2 [-14.4, -4]
Trauma and fractures	10.3 [-25, 45.6]	-38.9 [-43.7, -34]	-20.6 [-31.9, -9.3]	-16.1 [-19.4, -12.9]	-2.8 [-9.1, 3.5]	-20.3 [-26.3, -14.4]	-41.2 [-43.4, -39]	-49.9 [-53.9, -45.9]	-27.6 [-37.8, -17.4]	-14.7 [-17.4, -12.1]	-19.4 [-23.8, -15]
**Total consultations**	13 [-10.7, 36.8]	-43.5 [-50.7, -36.4]	-34.1 [-42, -26.2]	-18.9 [-24, -13.8]	-26.1 [-28.6, -23.7]	-33.2 [-37.5, -28.9]	-41.2 [-45.6, -36.9]	-54.9 [-58.3, -51.6]	-9.7 [-33.8, 14.4]	2.9 [-19, 24.9]	-16.8 [-18.3, -15.3]

Supplementary References

1. Moyes J, Walaza S, Chikosha S, et al. Epidemiology of respiratory pathogens from influenza-like illness and pneumonia surveillance programmes, South Africa, 2018. Natl Inst Commun Dis Bull **2019**; 17:36–60.
2. Tempia S, Walaza S, Bhiman JN, Mcmorrow ML, Moyes J, Mkhencele T. Decline of influenza and respiratory syncytial virus detection in facility-based surveillance during the COVID-19 pandemic, South Africa, January to October 2020. Eurosurveillance **2021**; 26:pii=2001600.
3. Google COVID-19 Community Mobility Reports. Available at: https://www.google.com/covid19/mobility/. Accessed 31 December 2021.
4. Hale T, Angrist N, Goldszmidt R, et al. A global panel database of pandemic policies (Oxford COVID-19 Government Response Tracker). Nat Hum Behav **2021**; 5:529–538.
5. Trapletti A, Hornik K. tseries: Time Series Analysis and Computational Finance. R Package version 010-42 **2017**;
6. Olsen SJ, Azziz-Baumgartner E, Budd AP, et al. Decreased influenza activity during the COVID-19 pandemic—United States, Australia, Chile, and South Africa, 2020. Am J Transplant **2020**; 20:3681–3685.
